# Diagnosed After Birth—But Detectable Before? A Cohort Study of Prenatal Testing Potential

**DOI:** 10.1002/pd.70072

**Published:** 2026-01-23

**Authors:** Allison Schartman, Olivia Woods, Leah Wetherill, Amy M. Breman, Benjamin M. Helm, Kristen Suhrie, Kristyne Stone

**Affiliations:** ^1^ Department of Medical and Molecular Genetics Indiana University School of Medicine Indianapolis Indiana USA; ^2^ Department of Pediatrics Division of Neonatal‐Perinatal Medicine Indiana University School of Medicine Indianapolis Indiana USA

## Abstract

**Objective:**

To evaluate the yield of prenatal genetic testing in infants with a confirmed genetic diagnosis.

**Methods:**

We retrospectively reviewed records of infants with a genetic diagnosis who were evaluated using a standardized genetic consult and testing approach. The predicted yield of various prenatal genetic sceening and diagnostic tools in this cohort was determined and compared.

**Results:**

Genome sequencing had the highest predicted diagnostic yield (96.9%), followed by CMA with reflex to exome sequencing (95.5%), exome sequencing alone (93.8%) and CMA alone (43.6%). ACOG‐recommended NIPT and carrier screening could have detected 25.4% of diagnoses, while 55.3% were detectable through genome‐wide NIPT and a large carrier screening panel. Genome‐wide NIPT improved chromosomal abnormality detection by ∼30% compared with ACOG‐recommended NIPT. A large commercial carrier screening panel detected 26.1% of single‐gene conditions, versus 6.1% with the ACOG‐recommended panel. Overall, 62% of single‐gene conditions were undetectable with current screening tools.

**Conclusion:**

Prenatal ES or GS offers high diagnostic yields and a streamlined approach, suggesting that CMA may not be the most appropriate first‐line test unless there is strong suspicion of a chromosomal diagnosis. Although prenatal genetic screening is valuable, its ability to identify rare genetic conditions remains limited. Our findings support revising the ACOG/ACMG guidelines to align with postnatal testing recommendations, particularly in high‐risk pregnancies.

## Summary

- What's already known about the topic?
  ◦ Previous studies have assessed the diagnostic yield of various prenatal genetic screening and diagnostic tools (NIPT, carrier screening, CMA, exome sequencing, genome sequencing) to determine the proportion of genetic diagnoses in a fetal cohort.
  ◦ Most studies focus on individual tests in the general prenatal population or in fetuses with a specific congenital anomaly, therefore providing an incomplete picture of the overall potential of prenatal genetic testing in infants with genetic conditions.
- What does this study add?
  ◦ Using a cohort of infants with confirmed genetic diagnoses, we compared all genetic testing options available in the prenatal setting to evaluate whether these diagnoses could have been made prenatally, and by which tests.
  ◦ Our data suggests the need for updated prenatal guidelines to reflect the increased diagnostic yield of exome and genome sequencing compared to the currently recommended CMA as a first‐line test when a fetal anomaly is identified.

## 1 Introduction

Rapid genome sequencing (GS) is increasingly being performed on infants in intensive care units (ICUs) with diagnostic yields ≥ 40% [1, 2, 3]. The utility of GS has been demonstrated via reduced costs (e.g., reduced length of admission) and changes to medical care [1, 3, 4]. Shifting the diagnosis to the prenatal period would be ideal, as accurate identification of genetic conditions before birth can significantly impact pregnancy management, delivery planning, and neonatal care [2, 5, 6]. Chromosome analysis (i.e., karyotype), fluorescence in situ hybridization (FISH), chromosomal microarray (CMA), exome sequencing (ES) and GS can all be performed on chorionic villi or amniocytes, enabling accurate prenatal diagnosis for many genetic conditions. Prenatal screening options have evolved significantly; non‐invasive prenatal testing (NIPT) and carrier screening are increasingly utilized to assess risk of chromosomal abnormalities and select genetic conditions.

Despite these advances, the diagnostic yield and comparative performance of current prenatal screening and diagnostic tools remain incompletely characterized, particularly in real‐world settings involving infants diagnosed with genetic conditions. Current guidelines from the American College of Obstetricians and Gynecologists (ACOG) and the American College of Medical Genetics and Genomics (ACMG) recommend CMA as the first‐line diagnostic test when a fetal anomaly is identified [7, 8]. However, the diagnostic yield of CMA alone is limited, ranging from 5% to 15%, depending on the specific fetal anomaly [9, 10, 11]. Comprehensive testing using GS has the potential to improve early detection by simultaneously identifying both chromosomal and monogenic disorders.

Previous studies have assessed the diagnostic yield of various prenatal genetic screening and diagnostic tools, including NIPT, carrier screening, CMA, ES, and GS [12, 13, 14, 15, 16, 17, 18]. Most research focuses on the yield of individual tools in the general prenatal population, sometimes in fetuses with specific ultrasound findings [12, 13, 14, 18, 19]. The performance of these prenatal tools in neonates with a confirmed genetic diagnosis has not been studied. For a cohort of hospitalized infants diagnosed with genetic conditions, this study examined the theoretical diagnostic yield of prenatal genetic testing, assuming that every patient had access to all available testing modalities.

## 2 Methods

### 2.1 Participants and Procedures

This primarily descriptive retrospective cohort study protocol was approved by the Indiana University (IU) Institutional Review Board (IRB‐04:23,594). Clinical data were collected and managed using Recap electronic data capture tools hosted at IU [20, 21]. REDCap (Research Electronic Data Capture) is a secure, web‐based software platform designed to support data capture for research studies. Data included demographics, timing of diagnosis (prenatal vs. postnatal), gestational age at birth, location of birth (Riley Children's Hospital vs. an outside hospital with transfer to Riley), and genetic test results with associated genetic variants and diagnoses.

Patients were identified from a clinical database maintained by the Department of Medical and Molecular Genetics at IU Health, as well as a research database (IRB#14499). These databases include patients admitted to the NICUs at Riley Children's Hospital at IU Health as well as other patients evaluated by the Medical Genetics inpatient consultative service.

An institutional algorithm (Supporting Information S1: Figure S1 and Table S1) was followed for patients in the NICUs to determine if a patient should receive a clinical genetics evaluation and to guide the genetic testing approach [22]. When a child in the NICUs presents with symptoms of a genetic condition (e.g., congenital anomaly and/or unexplained metabolic or organ dysfunction), the inpatient genetics team is consulted. The only exception is for infants known to have Down syndrome, for whom the developmental medicine service is consulted. Based on clinical evaluation, genetic testing may be deferred if symptoms can be explained by a non‐genetic etiology. When a specific condition is suspected or there is a known family history of a genetic condition, targeted testing is performed. For cases where the differential diagnosis is broad/uncertain, short‐read, rapid genome sequencing is performed following parental consent. At our institution, all inpatient genetic testing is ordered by the inpatient genetics service, except for karyotypes in babies with Down syndrome. Our standardized approach to consultation and testing ensures that all infants in the NICUs have equitable access to genetic testing, including GS.

Patients were included in the study if they were evaluated by the inpatient genetics service or developmental medicine (for infants with Down syndrome) at Riley Children's Hospital between July 2022 and July 2024 and diagnosed with a genetic condition prior to 1 year of life. Patients with only secondary findings or positive familial variant testing without symptoms at the time of testing were excluded [22]. A retrospective chart review was conducted for all patients to supplement missing clinical information and to provide additional details regarding genetic test results and the clinical interpretation of results.

### 2.2 Prenatal Testing for Detection of Genetic Conditions

For each patient, we determined which prenatal screening tools could have indicated an increased risk for their known genetic diagnosis, assuming that every patient had access to all available screening tools regardless of the indication or presence of fetal anomalies. We included various commercially available NIPT products that screen for trisomies 13, 18, 21 and common sex chromosome aneuploidy (“NIPT + SCA”); aneuploidy and common microdeletions (“NIPT + SCA + deletions”; deletions included 22q11, 15q11, 11q23, 8q24, 5p15, 4p16, 1p36); aneuploidy, common microdeletions and CNVs greater than 7 Mb (“genome‐wide NIPT”); and dominant single gene conditions (“single gene NIPT”) [17]. We assumed that a condition could be detected if screening via NIPT was validated to detect the condition and, for single gene NIPT, we assumed that the mother was unaffected by the condition. Carrier screening panels were also assessed, and we predicted that a condition could be identified if both parental variants could be detected for an autosomal recessive disorder or if a maternal variant could be detected for an X‐linked condition. We differentiated among carrier screening panels recommended by ACOG and ACMG (“ACOG carrier screen” and “ACMG carrier screen”, respectively) as well as a large commercially available panel. The ACOG carrier screening included cystic fibrosis, spinal muscular atrophy and hemoglobinopathies/thalassemias [23]. The ACMG carrier screen included 113 Tier 3 conditions with > 1/200 carrier frequency and specific X‐linked conditions [24]. The commercial panel (“commercial carrier screening panel”) was created by combining two current commercial panels with 613 conditions and 267 genes; the total number of unique genes was 614 [25, 26]. See (Supporting Information S3: Tables S1–S3) for details on each carrier screening panel. Single gene NIPT for autosomal recessive conditions was not included, as the same conditions are included in carrier screening, which is more comprehensive. The NIPT and carrier screening tools selected for each diagnosis were not mutually exclusive.

We also determined which specific diagnostic prenatal tests (FISH, karyotype, CMA, ES, GS, other) could have detected each condition using chorionic villus sampling (CVS) and/or amniocentesis, assuming that every patient had access to all available testing modalities regardless of whether fetal anomalies were present. Test selection was based on GS capabilities/reporting through the laboratory utilized by our institution at the time of the study. Notably, this laboratory did not detect the homozygous SMN1 deletion associated with SMA. When multiple tests were capable of diagnosing a condition, all applicable options were included.

To ensure the accuracy of the prenatal screening and diagnostic tests selected, two genetic counselors with over 30 years combined experience in prenatal genetic counseling (A.S., K.S.) reviewed the test selection for every diagnosis. In instances where rare or unusual variants were identified, an expert board‐certified in clinical laboratory cytogenetics and molecular genetics/genomics (A.B.) confirmed the test selection.

Each diagnosis was coded as primary, incidental, or targeted familial variant. A primary diagnosis explained the infant's phenotype at the time of testing, whereas an incidental finding was associated with a clinically significant childhood onset condition in the absence of symptoms at the time of testing.

Complex diagnoses were evaluated as follows. For unbalanced rearrangements, if either the deletion or the duplication was > 7Mb, it was categorized as detectable via genome‐wide NIPT even if both copy number variants may not be detected. We reviewed the genetic tests performed for each patient and then assessed which of the available prenatal testing strategies would be expected to detect mosaic findings in a fetal sample.

Variants were classified as follows. Patients with a VUS (per the genetic testing laboratory) that was considered clinically diagnostic by the medical geneticist were included in the cohort, although these may not always be reported prenatally. For prenatal screening tool selection, if a variant was classified as a VUS by the laboratory, it was categorized as “no screening available” since VUSs are not likely to be reported on carrier screening or single gene NIPT. Conditions with intronic variants were categorized as “no screening available,” as most carrier screening tests cannot detect deep intronic variants (apart from some common intronic variants in certain genes, e.g., *HBB*). For prenatal diagnostic testing options, when one or both variants were classified by the lab as a VUS, we based the categorization on the diagnostic tool's ability to detect the variant. For autosomal recessive conditions, if one of the two variants was detectable only by GS, GS was selected as the only diagnostic test able to detect that condition since a definitive diagnosis would not be possible without GS.

### 2.3 Analysis

#### 2.3.1 Descriptive Statistics

We summarized the proportion of diagnoses identified by the various prenatal tests. To evaluate the predicted yield of each test, we summed across all the diagnoses selected for the specific test and divided by the total number of diagnoses identified in the cohort (*n* = 291). We used the total number of diagnoses (including diagnoses occurring in multiple patients) to capture the overall yield of each test in this real‐world cohort. While diagnostic yield is often defined as the “likelihood that a test or procedure will provide the information needed to establish a diagnosis”, for this study, the “yields” reported represent the proportion of genetic diagnoses that could be made by a specific prenatal test to allow for comparison of each testing method in this population [27].

## 3 Results

### 3.1 Study Cohort

A total of 281 patients met the criteria for study inclusion. Table 1 details demographic information for the entire cohort. The cohort was majority male at birth (150, 53.4%), white (203, 72.2%), non‐Hispanic (236, 84%), > 37 weeks gestational age (177, 63%), and inborn at Riley Children's Hospital (151, 53.7%).

**TABLE 1:** Demographic information.

	*N* = 281
Sex assigned at birth	
Male	150 (53.4%)
Female	131 (46.6%)
Race	
White	203 (72.2%)
Black	48 (17.1%)
Asian	6 (2.1%)
Native american or Alaskan native	1 (0.4%)
Other	12 (4.3%)
Not reported	12 (4.3%)
Ethnicity	
Not hispanic	236 (84%)
Hispanic	39 (13.9%)
Not reported	6 (2.1%)
Location of birth	
Riley children's hospital (inborn)	151 (53.7%)
Outside facility	130 (46.3%)
Gestational age at birth	
> 37 weeks	177 (63%)
34–36 weeks	41 (14.6%)
28–34 weeks	54 (19.2%)
< 28 weeks	7 (2.5%)
Not reported	2 (0.7%)

**FIGURE 1:**
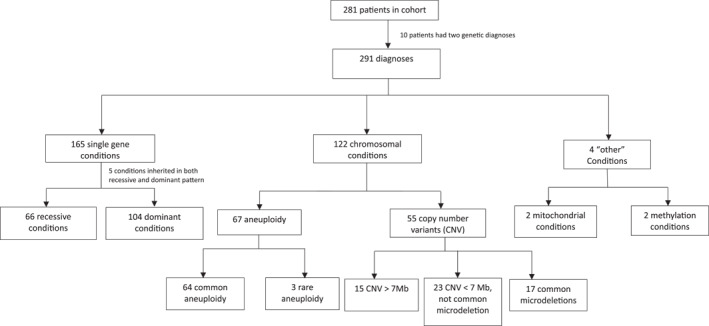
Breakdown of conditions observed in the study cohort. Common microdeletion syndromes included were 22q11, 15q11,11q23, 8q24, 5p15, 4p16, 1p36.

Across the study cohort, 336 diagnostic tests were performed, either prenatally or during the inpatient stay, resulting in 291 diagnoses, 259 (89%) primary, 24 (8.2%) incidental and 8 (2.7%) known familial variants. Ten (3.6%) of the 281 patients had two genetic diagnoses. There were 181 unique genetic diagnoses. Trisomy 21, diagnosed in 48 patients, was the most common diagnosis in the cohort. Rapid GS was the most common test performed, ordered for 170 (60.5%) patients. The majority of patients (154, 90.4%) received a genetic diagnosis after birth, while 9.6% (27) were diagnosed prenatally. Figure 1 summarizes the types of genetic conditions in the cohort: 165 single gene conditions, 122 chromosomal abnormalities (67 aneuploidies and 55 copy number variants), and 4 more rare types of diagnoses. (Supporting Information S2: Table S1) details the specific diagnoses and test(s) selection.

### 3.2 Predicted Prenatal Diagnostic Testing Yield

**FIGURE 2:**
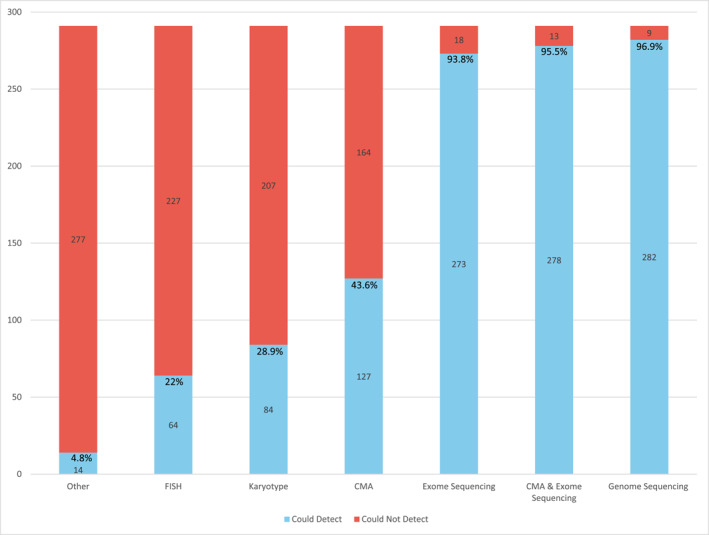
Predicted yield of prenatal diagnostic tools for the entire study cohort (*n* = 291). Blue bars represent the number of diagnoses that each diagnostic tool could have identified, while the red bars represent the number of diagnoses that each tool could not have identified. Percentages are shown to indicate the proportion of the sample that could have been identified by the prenatal test(s). Other testing includes diagnoses that can be made through methylation testing, targeted gene analysis, and southern blot. CMA = chromosomal microarray; FISH = fluorescence in situ hybridization for T21, T13, T18 and sex chromosome aneuploidy.

Figure 2 demonstrates the predicted yield of various prenatal diagnostic tests in the cohort of neonates with a genetic diagnosis. Of the 291 diagnoses, 282 (96.9%) were detectable with GS, 273 (93.8%) with ES, and 127 (43.6%) with CMA. CMA with reflexive ES (when CMA normal) could have detected 278 (95.5%) diagnoses in the cohort, requiring two different diagnostic tests to achieve a diagnosis. Of the total diagnoses, 9 (3.1%) could not have been detected by GS. Notably, if GS that captures the common homozygous SMN1 deletion were utilized, the number of diagnoses not detected by GS would only be 5 (1.7%). Additionally, two patients in the cohort were diagnosed with Beckwith‐Weidemann syndrome via methylation studies on amniotic fluid. CMA and deletion/duplication of IC1 and IC2 were negative for these individuals; however, additional molecular testing (ES or GS) was not performed.

The diagnostic tests selected for each unique diagnosis are outlined in Supporting Information S2: Table S1. Four conditions could be diagnosed via GS but not CMA with reflexive ES. These conditions included three deep intronic variants and myotonic dystrophy type 1. Of these, two deep intronic variants could have been detected exclusively via GS, and two could be detected via other methodologies if performed (i.e., myotonic dystrophy and *HBB* sequencing). Of the 9 diagnoses that could not have been detected via GS, two required methylation studies, five required targeted gene analysis and 2 were mitochondrial DNA variants for which prenatal testing is currently unavailable in the United States.

### 3.3 Predicted Prenatal Screening Tests Yield for Entire Cohort

**FIGURE 3:**
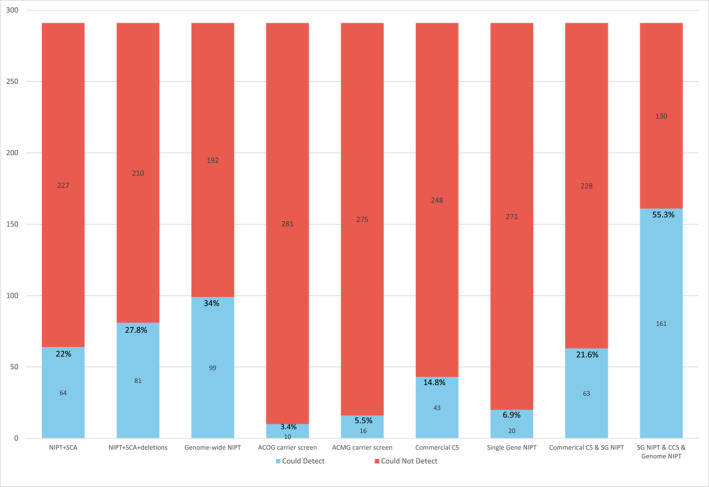
Predicted yield of various screening tools for the entire study cohort (*n* = 291), assuming 100% sensitivity. The blue bars represent the number of diagnoses in the cohort that could be detected by each screening tool, while the red bars represent the number of diagnoses in the cohort that could not be detected. Percentages are shown to indicate the proportion of the sample that could have been identified by the prenatal screen(s). ACOG = American College of Obstetrics and Gynecology; ACMG = American College of Medical Genetics and Genomics; CS = carrier screening; CCS = commercial carrier screening; NIPT = non‐invasive prenatal testing; SCA = sex chromosome aneuploidy; SG = single gene.

Of the 291 diagnoses, 161 (55.3%) could be identified through commercially available NIPT or carrier screening, while 130 (44.7%) could not be identified by any available prenatal screening tool. If traditional NIPT (NIPT + SCA) and ACOG‐recommended carrier screening were performed for the cohort (consistent with ACOG guidelines), only 74 (25.4%) diagnoses could have been detected prenatally via one of these tools. The predicted yield of each available screening tool in this cohort of neonates with a genetic diagnosis is detailed in Figure 3.

### 3.4 Screening for Chromosome Abnormalities

**FIGURE 4:**
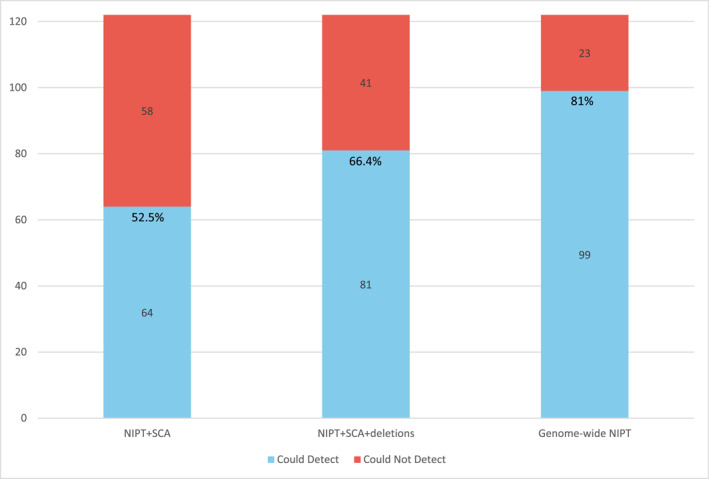
Predicted yield of various NIPTs for chromosomal diagnoses in the study cohort (*n* = 122). The blue bars represent the number of diagnoses that could be detected by the chosen NIPT, while the red bars represent the number of diagnoses that could not be detected. Percentages are shown to indicate the proportion of the sample that could have been identified by the prenatal screening. NIPT = non‐invasive prenatal testing; SCA = sex chromosome aneuploidy.

The predicted yield of various chromosomal screening tools in this cohort is detailed in Figure 4. For chromosomal diagnoses (*n* = 122), genome‐wide NIPT had the highest predicted yield (99, 81.1%), followed by NIPT + SCA + deletions (81, 66.4%) and NIPT + SCA (64, 52.5%). There were 23 (19%) chromosomal diagnoses that could not be detected by current prenatal NIPT options.

### 3.5 Screening for Single‐Gene Conditions

**FIGURE 5:**
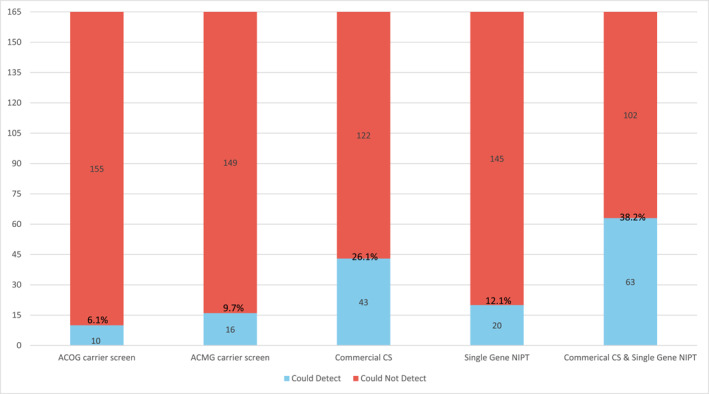
Predicted yield of various screening options for the single gene conditions in the study cohort (*n* = 165). The blue bars represent the number of diagnoses that could be detected, while the red bars represent the number of diagnoses that could not be detected. Percentages are shown to indicate the proportion of the sample that could have been identified by the prenatal screen(s). Commercial CS = commercial carrier screening; NIPT = non‐invasive prenatal testing.

The yield of various prenatal single‐gene screening tools in this cohort is outlined in Figure 5. Of the 165 single‐gene conditions, ACOG carrier screening could detect 10 (6.1%), ACMG carrier screening could detect 16 (9.7%), and the commercial carrier screening panel could detect 43 (26.1%). Of the 66 recessive conditions, the commercial carrier screening panel had the highest yield (43, 65.2%), followed by ACMG carrier screening (16, 24.2%) and ACOG carrier screening (10, 15.1%). Among the 104 dominant conditions, 20 (19.2%) could be detected by commercially available single‐gene NIPT. Most of the single‐gene conditions (102/165, 61.8%) could not be detected by any commercially available prenatal screening tool (carrier screening or single gene NIPT).

### 3.6 Additional Considerations

Of all diagnoses (291), 10 (3.4%) involved a chromosomal rearrangement, 5 (1.7%) had multiple inheritance patterns (i.e., dominant and recessive), and 11 (3.8%) involved a VUS that was clinically considered diagnostic. The VUSs included 7 recessive conditions where one or both variants were classified as a VUS and 4 dominant conditions where a single variant was classified as VUSs. Among the 66 recessive conditions in the sample, 3 had one variant that could only be identified via GS. Of the 122 chromosomal diagnoses, 5 were mosaic. The complexity of these diagnoses made it difficult to select the prenatal screening tool and/or predict the yield of a screening or diagnostic tool as detection/reporting may vary between laboratory or other factors (e.g., specimen type).

## 4 Discussion

### 4.1 Prenatal Diagnostic Testing

Even though 97% of genetic conditions necessitating inpatient tertiary care were detectable with GS and 94% were detectable with ES, only 10% of patients in this study received a prenatal diagnosis. Shifting the diagnosis to the prenatal period would allow for improved patient counseling and decision making, as well as better‐informed pregnancy and neonatal management [1, 2, 5, 6, 28]. However, since genomic sequencing is currently limited to pregnancies with fetal anomalies, and accurate variant interpretation relies on standardized HPO‐based phenotypic descriptions, adopting it as a true first‐line test would require careful consideration.

Multiple studies support the use of rapid GS in inpatient/acute postnatal settings and ACMG and the American Academy of Pediatrics strongly recommend ES/GS be considered as first‐line testing in pediatric patients with congenital anomalies or intellectual disability/global developmental delay; however, prenatal guidelines still recommend CMA as first‐line testing when a fetal anomaly is diagnosed [1, 28, 29, 30]. If CMA is negative, ACOG suggests targeted molecular testing; ACMG recommends considering ES [7, 8]. Based on this guidance, health insurance payors often require CMA to be performed prior to approving coverage for prenatal ES [31]. This increases the time to diagnosis, which is critical in the prenatal setting. In a cohort of infants with genetic disease, this study provides evidence that prenatal GS provides the highest diagnostic yield without requiring reflexive testing associated with delayed diagnosis, increased cost, and additional sample requirements. However, prenatal GS is not currently a covered benefit, so the cost is prohibitive for most families. It is also important to note that ES/GS are phenotypic‐driven analyses, and it is unclear how much of the neonatal phenotype in our cohort could have been known prenatally. GS has been used in an asymptomatic newborn population with reports of predefined disease‐causing variants [32, 33]. These studies have yet to be performed in a fetal population; however, a similar approach is theoretically feasible in low‐risk pregnancies and may provide information on the utility of ES/GS in non‐anomalous pregnancies, which was not evaluated in the current study.

This study also highlights some limitations of GS. In our cohort, 3% of diagnoses required targeted or specialized testing. Notably, four cases of spinal muscular atrophy (SMA) involving pathogenic variants in the *SMN1* gene were identified through methods other than GS. This underscores both the value of parental carrier screening, as well as the ongoing challenges in detecting certain variant types that may be missed by both prenatal ES and GS (depending on the specific bioinformatics tools utilized by the laboratory). In addition, certain variants associated with *HBB*‐related hemoglobinopathies/thalassemias and repeat disorders (e.g., fragile *X* syndrome) would be missed by ES but detected via carrier screening. This data also highlights the limitations of prenatal ES and GS for conditions that require methylation analysis, conditions with pseudogenes (e.g., IKBKG‐associated incontinentia pigmenti), and mitochondrial conditions. Finally, 4% of diagnoses in our cohort involved a VUS that was interpreted as diagnostic by the clinical teams. In the prenatal setting, VUSs are less likely to be reported and are challenging to interpret given the limited phenotypic data available to prenatal providers.

As the field of genomics continues to rapidly evolve and expand, many clinicians struggle to remain current in their knowledge of fetal genetic conditions and associated screening and diagnostic approaches. A recent survey identified a critical gap in knowledge among 100 non‐genetic prenatal care clinicians regarding these challenges, with 39% reporting feeling confident or extremely confident in their knowledge. However, among those clinicians, the majority were unable to answer all ACOG knowledge questions correctly [34]. Knowledge regarding prenatal genomic sequencing technology is likely to be even more limited. This knowledge gap, as well as results from our study regarding the limitations of genomic sequencing and the nuances of test/laboratory selection, emphasizes the importance of involving genetics providers in prenatal genetic test selection, results interpretation, and counseling families about associated benefits and limitations.

### 4.2 Prenatal Screening Tools

While prenatal diagnostic testing has the highest likelihood of providing a fetal diagnosis, families may decline CVS or amniocentesis because of procedure‐associated risks. Since families may opt for screening in lieu of diagnostic prenatal testing, we also compared the yield of various prenatal screening tools in our cohort of neonates with a confirmed genetic diagnosis. It is important to note that the yield of these tools in this population does not reflect the yield in a low‐risk prenatal population.

If ACOG recommendations for NIPT and carrier screening were strictly followed, only one‐quarter of the genetic diagnoses in our cohort could have been detected prenatally (assuming no false negative results). The addition of all commercially available screening options increased detection to around 55%. The fact that over 40% of all genetic diagnoses (and 62% of monogenic conditions) would be missed with the most comprehensive screening approach highlights the limitations of screening and the benefit of diagnostic testing with genomic sequencing, particularly in pregnancies with known fetal anomalies or other concerns.

We compared the yield of various NIPT options in identifying chromosomal abnormalities. The use of genome‐wide NIPT resulted in an approximate 30% increase in yield compared with ACOG‐recommended NIPT. Despite this increased yield, there is a debate about whether to offer genome‐wide NIPT for low‐risk pregnancies. Concerns include the increased identification of chromosome abnormalities with uncertain clinical consequences (e.g., due to confined placental mosaicism, etc.) and lower positive predictive values (e.g., associated with rare autosomal trisomies, CNVs, etc.). These challenges are among the reasons why ACMG and ISPD argue against the broader use of expanded NIPT [35, 36]. This study also underscored the benefits of expanded carrier screening compared with ACOG‐recommended carrier screening, while simultaneously demonstrating the overall limited utility of prenatal screening tools for the detection of single‐gene conditions. Prospective parents considering more comprehensive NIPT and carrier screening would benefit from comprehensive pre‐ and post‐test counseling by a genetics specialist; however, this is likely not feasible in the general prenatal population, highlighting the need for further genomic education for obstetric providers.

### 4.3 Strengths, Limitations, and Future Research

This study included a large cohort of critically ill infants with confirmed genetic diagnoses following a comprehensive standardized approach to genetic evaluation and testing. Our methods allowed direct comparison of each prenatal testing option to determine which tests had the highest predicted yield. However, it is possible that some infants with a genetic condition were undiagnosed and not included in our cohort because of limitations of current genetic testing or because they were not symptomatic/severe enough to be hospitalized and/or fit our algorithm for consultation in the NICU. Genetic conditions presenting in later infancy, childhood, or even adulthood were not evaluated in this study, but prenatal knowledge of these genetic conditions is also likely important to expectant parents and should be considered for future research. Additionally, as our cohort was made up of those with a genetic condition, our results do not apply to general population screening/testing.

Our approach to selecting prenatal screening and diagnostic tools may have overestimated the yield since we assumed a sensitivity of 100% and did not account for whether dominant conditions were inherited or *de novo*. Additionally, we assumed that all testing would be offered and available. Test offerings/strategies vary widely depending on the provider and clinical scenario, and prenatal GS is currently not widely available or covered by insurance. Detection of specific diagnoses, such as SMA and mosaic aneuploidy, can also vary depending on laboratory methodology and, for cases of mosaicism, specimen type.

This study did not collect data on associated fetal ultrasound findings or other prenatally identified concerns, as the goal of this study was to determine the potential yield of specific prenatal tests, regardless of why they are performed or whether an anomaly was identified. Moreover, anomalies are detected in approximately 3%–5% of pregnancies and are a common reason individuals pursue diagnostic genetic testing or additional prenatal screening [37]. When a fetal risk is known prenatally, delivery at Riley Children's Hospital is typically recommended for access to higher‐level care and genetic testing. A little less than half of the births in our cohort occurred outside Riley, suggesting that these pregnancies did not have a known fetal risk. This may imply that many of the diagnoses in our cohort did not have an identified fetal anomaly/concern, which may have prompted the consideration of more comprehensive genetic testing. Future research would be beneficial to determine the potential yield of comprehensive screening and diagnostic testing in specific clinical settings (e.g., fetus with multiple anomalies vs. isolated anomaly vs. low‐risk pregnancy) and evaluate the utility of ES/GS in non‐anomalous pregnancies.

## 5 Conclusion

This study evaluates the diagnostic yield and comparative performance of current prenatal screening and diagnostic tools in a cohort of critically ill infants with genetic conditions. It emphasizes the nuances of prenatal genetic testing options currently available and the need for experienced providers to be involved in test selection, result interpretation, and pre‐ and post‐test counseling. The results highlight the limitations of prenatal screening, particularly when the chance of a fetal genetic condition is high, and the differential diagnosis is broad (such as a fetal anomaly). Unless a chromosomal diagnosis is strongly suspected, our findings suggest that CMA should no longer be considered the first‐line test when the chance of a genetic condition is high—such as in the setting of a fetal anomaly—because GS offers higher yield and provides a comprehensive single‐step analysis. It also underscores the need for revision of ACOG guidelines to align with postnatal testing recommendations and to facilitate reimbursement for comprehensive prenatal genomic sequencing.

## Funding

The authors have nothing to report.

## Ethics Statement

This study protocol was approved by the Indiana University (IU) Institutional Review Board (IRB‐04:23,594) on July 3, 2024.

## Consent

Patient consent was not applicable for this study because of the retrospective nature of the study and the use of anonymized clinical data.

## Conflicts of Interest

Kristen Suhrie, MD reports receiving an honorarium from Illumina for a speaking engagement in 2023. All other authors declare no conflicts of interest.

## Supporting information


Supporting Information S1

Supporting Information S2

Supporting Information S3

## Data Availability

The data that support the findings of this study are available on request from the corresponding author. The data are not publicly available due to privacy or ethical restrictions.
