# Supplementary material for: Diagnosed After Birth—But Detectable Before? A Cohort Study of Prenatal Testing Potential
Source: Prenat Diagn. 2026 Jan 23;46(5-6):904–13. doi: 10.1002/pd.70072 (PMC13170046; doi:10.1002/pd.70072)
Supplement: Supplementary file 1 — Supporting Information S1 [file PD-46-904-s001.docx]

Supporting Information 1: Algorithm for provision of genetics services in the NICU


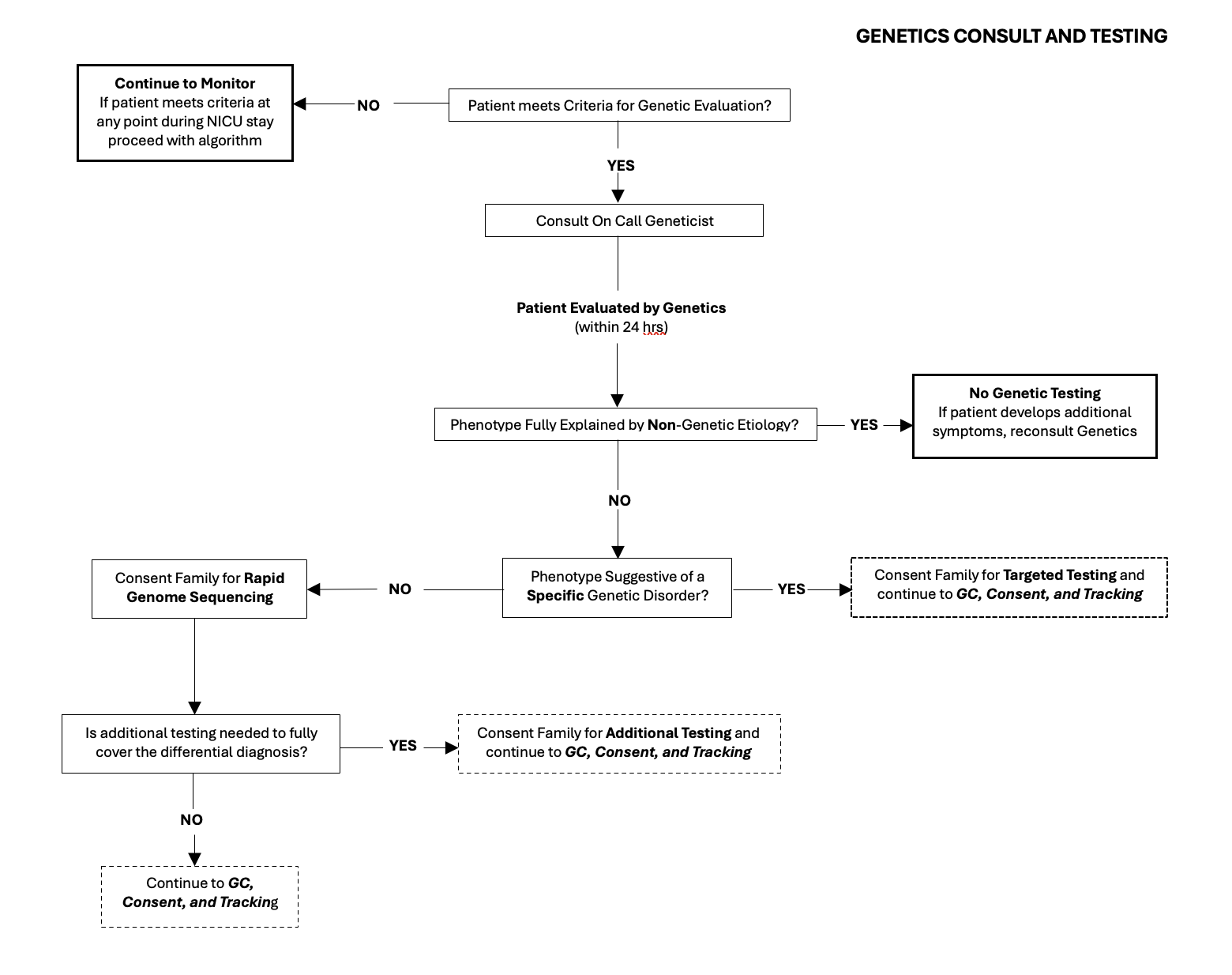


Figure S1: Algorithm for Provision of Genetics Services

Abbreviations: NICU=neonatal intensive care unit; GC=Genetic Counseling

Table S1: Genetic Evaluation Inclusion Criteria for Infants in the NICU

| Criteria | Examples |
| --- | --- |
| Congenital Anomalies, Dysmorphic Features, and/or Hydrops Fetalis | Congenital heart disease, brain anomalies, cleft palate |
| Unexplained Metabolic Abnormalities | Hyperammonemia, unexplained acidosis and/or electrolyte abnormalities |
| Cardiac Dysfunction | Cardiomyopathy, heart failure, abnormal cardiac valves |
| Unexplained Respiratory Symptoms | Respiratory failure, needing oxygen or positive pressure at term gestation not consistent with infection, delayed surfactant production |
| Unexplained Neurological Symptoms | Hypotonia, hypertonia, seizures, arthrogryposis, abnormal muscle mass |
| Unexplained Hematological Findings | Bleeding, clotting without a central line in place, indirect hyperbilirubinemia secondary to non-immune hemolysis, unexplained anemia, thrombocytopenia, WBC abnormalities |
| Evidence of Malignancy | Solid tumor or hematological malignancy |
| Evidence of Immune Dysfunction | Abnormal WBC, differential or subpopulations; infections not explained by prematurity, congenital anomaly, or foreign body (i.e. central line or foley catheter); infection with unusual organisms |
| GI Abnormalities and/or Dysfunction | Cholestasis, abnormal stooling, liver failure, hepatitis, pancreatic insufficiency |
| GU Abnormalities and/or Dysfunction | Abnormal renal function, abnormal urine quality or quantity |
| Bone or Joint Abnormalities | Unexplained fractures, missing or hypoplastic bones, bone deformities |
| Abnormal Skin Findings (not related to a benign newborn condition) | Abnormal pigmentation, unexplained blistering, nail abnormalities |
| Endocrine Dysfunction (not related to isolated hypothyroidism) | Hyperinsulinemia, pituitary dysfunction, adrenal dysfunction |
| Evidence of Systemic Inflammation (with or without joint involvement) | Unexplained fevers, evidence of inflammation without infection, joint swelling |
| Developmental or Constitutional Delay | Unexplained feeding difficulties at term, failure to thrive, abnormal responsiveness |
