# Supplementary material for: Diagnosed After Birth—But Detectable Before? A Cohort Study of Prenatal Testing Potential
Source: Prenat Diagn. 2026 Jan 23;46(5-6):904–13. doi: 10.1002/pd.70072 (PMC13170046; doi:10.1002/pd.70072)
Supplement: Supplementary file 2 — Supporting Information S2 [file PD-46-904-s002.docx]

Supporting Information 2: Unique diagnoses observed in cohort and prenatal test selections capable of detection

| **Diagnosis Name** | **Chromosome/ Gene** | **Variant** | **Diagnostic Test** | **Screening Test** |
| --- | --- | --- | --- | --- |
| **Chromosome Abnormalities** | | | | |
| 1p36 del (1p36.33p36.31) | 1 | 1p36.33p36.31 del | CMA, ES, GS | NIPT+SCA+deletion, Genome-wide NIPT |
| 1q21.1 del (1q21.1q21.2) (n=4) | 1 | 1q21.1q21.2 del | CMA, ES, GS | None |
| 1q21.1 dup (1q21.1q21.2) (n=2) | 1 | 1q21.1q21.2 dup | CMA, ES, GS | None |
| 2p21p12 dup | 2 | 2p21p12 dup | Karyotype, CMA, ES, GS | Genome-wide NIPT |
| 2q37.1q37.3 del | 2 | 2q37.1q37.3 del | Karyotype, CMA, ES, GS | Genome-wide NIPT |
| 3p26.3p14.3 dup | 3 | 3p26.3p14.3 dup | Karyotype, CMA, ES, GS | Genome-wide NIPT |
| 4q12 del | 4 | 4q12 del | CMA, ES, GS | None |
| Wolf-Hirschhorn syndrome (n=2) | 4 | 4p16.3p15.2 del  4p16.3p15.33 del | Karyotype, CMA, ES, GS | NIPT+SCA+deletion, Genome-wide NIPT |
| Cri-du-Chat syndrome | 5 | 5p15.33p14.3 del | Karyotype, CMA, ES, GS | NIPT+SCA+deletion, Genome-wide NIPT |
| Chromosome 7 rearrangement | 7 | 7p22.3p14.3 dup; 7q22.3q31.1 del | Karyotype, CMA, ES, GS | Genome-wide NIPT |
| Williams-Beuren Syndrome | 7 | 7q11.23 del | CMA, ES, GS | None |
| Chromosome 8 rearrangement | 8 | 8pterp23.2 del; 8q24.22q24.23 del; 8q21.13q24.13 dup; 8q24.23qter dup | Karyotype, CMA, ES, GS | Genome-wide NIPT |
| 8p23q21.3 dup | 8 | 8p23q21.3 dup | Karyotype, CMA, ES, GS | Genome-wide NIPT |
| Mosaic trisomy 8 (Warkany syndrome) | 8 | Mosaic trisomy 8 | Karyotype, CMA, GS | Genome-wide NIPT |
| Unbalanced 5;9 translocation | 9 | 47,XX,+der(9)t(5;9)(p13;q21) | Karyotype, CMA, ES, GS | Genome-wide NIPT |
| 9p4.3p23 del | 9 | 9p4.3p23 del | Karyotype, CMA, ES, GS | Genome-wide NIPT |
| Mosaic trisomy 9 | 9 | Mosaic trisomy 9 | Karyotype, CMA, GS | Genome-wide NIPT |
| 9p24.3p13.1 dup | 9 | 9p24.3p13.1 dup | Karyotype, CMA, ES, GS | Genome-wide NIPT |
| 10q22.2q23.2 dup (n=2) | 10 | 10q22.2q23.2 dup | CMA, ES, GS | Genome-wide NIPT |
| Mosaic Pallister-Killian syndrome | 12 | 47,XX,+i(12)(p10)[5]/46,XX[33] | Karyotype, CMA, GS | Genome-wide NIPT |
| Ring chromosome 12 | 12 | 46,XX,r(12)(p13.3q24.33 | Karyotype, CMA, ES, GS | None |
| Trisomy 13 (n=3) | 13 | Trisomy 13 | FISH, Karyotype, CMA, ES, GS | NIPT+SCA, NIPT+SCA+deletions, Genome-wide NIPT |
| Kagami-Ogata syndrome | 14 | 14q32.2q32.33 | CMA, ES, GS, other-methylation | None |
| 15q11.2 del | 15 | 15q11.2 del | CMA, ES, GS | None |
| 15q13.2q13.3 del | 15 | 15q13.2q13.3 del | CMA, ES, GS | None |
| 15q25.1q25.2 del | 15 | 15q25.1q25.2 del | CMA, ES, GS | None |
| Angelman syndrome (n=2) | 15 | 15q11.2q13.1 del | CMA, ES, GS, other - methylation | NIPT+SCA+deletion, Genome-wide NIPT |
| Prader-Willi syndrome | 15 | 15q11.2q13.1 del | CMA, ES, GS, other - methylation | NIPT+SCA+deletion, Genome-wide NIPT |
| 16p11.2 del (n=2) | 16 | 16p11.2 del | CMA, ES, GS | None |
| 16p13.11 del | 16 | 16p13.11 del | CMA, ES, GS | None |
| Mosaic trisomy 16 | 16 | Mosaic trisomy 16 | Karyotype, CMA, GS | Genome-wide NIPT |
| 17q11.2 del | 17 | 17q11.2 del | CMA, ES, GS | None |
| Koolen-De Vries syndrome | 17 | 17q21.31 del | CMA, ES, GS | None |
| Miller-Dieker syndrome | 17 | 17p13.3p13.2 del | CMA, ES, GS | None |
| Smith-Magenis syndrome | 17 | 17p11.2 del | CMA, ES, GS | None |
| 18q22.1q23 del | 18 | 18q22.1q23 del | Karyotype, CMA, ES, GS | Genome-wide NIPT |
| Trisomy 18 (n=6) | 18 | Trisomy 18 | FISH, Karyotype, CMA, ES, GS | NIPT+SCA, NIPT+SCA+deletions, Genome-wide NIPT |
| Ring chromosome 18 | 18 | 46,XY,r(18)(p11.1q21.3) | Karyotype, CMA, ES, GS | Genome-wide NIPT |
| 19q13.33q13.4 3 dup | 19 | 19q13.33q13.4 3 dup | Karyotype, CMA, ES, GS | Genome-wide NIPT |
| 20q13.33 del | 20 | 20q13.33 del | CMA, ES, GS | None |
| Trisomy 21 (Down syndrome) (n=48) | 21 | Trisomy 21 | FISH, Karyotype, CMA, ES, GS | NIPT+SCA, NIPT+SCA+deletions, Genome-wide NIPT |
| 22q11.2 deletion syndrome (n=10) | 22 | 22q11.21 del | CMA, ES, GS | NIPT+SCA+deletion, Genome-wide NIPT |
| 22q11.2 dup | 22 | 22q11.21 dup | CMA, ES, GS | None |
| Klinefelter syndrome (n=2) | X | 47,XXY | FISH, Karyotype, CMA, ES, GS | NIPT+SCA, NIPT+SCA+deletions, Genome-wide NIPT |
| Trisomy X | X | 47,XXX | FISH, Karyotype, CMA, ES, GS | NIPT+SCA, NIPT+SCA+deletions, Genome-wide NIPT |
| Monosomy X (Turner syndrome) (n=2) | X | 45,X | FISH, Karyotype, CMA, ES, GS | NIPT+SCA, NIPT+SCA+deletions, Genome-wide NIPT |
| Mosaic Monosomy X | X | 46,X,add(X)(q28)[16]/45,X[14] | FISH, Karyotype, CMA, GS | NIPT+SCA, NIPT+SCA+deletions, Genome-wide NIPT |
| Xp11.23p11.22-related disorder | X | Xp11.23p11.22 | CMA, ES, GS | None |
| **Monogenic Conditions** | | | | |
| Achondroplasia (n=3) | *FGFR3* | c.1138G>A (p.G380R)  c.1138G>A (p.G380R)  c.1138G>A (p.G380R) | ES, GS | Single-gene NIPT |
| Achromatopsia 2 | *CNGA3* | c.847C>T (p.R283W) / c.830G>A (p.R277H) | ES, GS | None |
| Acyl-CoA dehydrogenase, short-chain deficiency of | *ACADS* | c.1058C>T (p.S353L) / c.1031A>G (p.E344G) | ES, GS | Commercial Carrier Screen |
| Adams-Oliver syndrome | *DOCK6* | c.5235+1_5235+7del | ES, GS | None |
| Alagille syndrome | *JAG1* | c.359T>C (p.I120T) | ES, GS | Single-gene NIPT |
| Alagille syndrome 2 | *NOTCH2* | c.5177G>A (p.R1726H) | ES, GS | None |
| Alport syndrome 1, X-linked | *COL4A5* | c.3721G>A (p.G1241S) | ES, GS | Commercial Carrier Screen |
| Arrhythmogenic right ventricular dysplasia 9 | *PKP2* | chr12:32861811-32873075 del (in GRCh38) | ES, GS | None |
| Arthrogryposis, distal, type 2A (Freeman-Sheldon syndrome) | *MYH3* | c.2015G>A (p.R672H) | ES, GS | None |
| Autosomal dominant Tetralogy of Fallot | *TBX1* | c.410+1G>C | ES, GS | None |
| Axenfeld-Rieger syndrome, Type 3 | *FOXC1* | c.388C>T (p.L130F) | ES, GS | None |
| Bardet-Biedl syndrome | *BBS5* | c.303dup (p.N102*) (homozygous) | ES, GS | Commercial Carrier Screen |
| Beckwith-Wiedemann syndrome | *CDKN1C* | c.*5+1G>A | ES, GS, other - methylation | None |
| Beckwith-Wiedemann syndrome† (n=2) | LIT1 | IC2 hypomethylation | other - methylation | None |
| Blepharocheilodontic syndrome 1 | *CDH1* | c.1107C>A (p.N369K) | ES, GS | None |
| Bone mineral density QTL18, osteoporosis | *PLS3* | c.234_237+10del | ES, GS | None |
| C8 Deficiency,  Type I | *C8A* | c.1152T>A (p.Y384*) (homozygous) | ES, GS | None |
| CACNA1C-Related Arrhythmia | *CACNA1C* | c.1216G>A (p.G406R) | ES, GS | None |
| Campomelic dysplasia | *SOX9* | c.326T>C (p.M109T) | ES, GS | None |
| Capillary malformation-arteriovenous malformation 1 | *RASA1* | c.1486del (p.Ile496Serfs*2) | ES, GS | None |
| Cardiofaciocutaneous syndrome 4 | *MAP2K2* | c.383C>G (p.P128R) | ES, GS | Single-gene NIPT |
| CDKL5-related developmental and epileptic encephalopathy | *CDKL5* | c.587C>T (p.S196L) | ES, GS | None |
| CHARGE syndrome (n=3) | *CHD7* | c.6955C>T (p.R2319C)  c.668del (p.Gly223Glufs*82) c.1898C>A (p.S633*) | ES, GS | Single-gene NIPT |
| Chung-Jansen syndrome | *PHIP* | c.2902C>T (p.R968*) | ES, GS | None |
| Ciliary dyskinesia, primary 3 | *DNAH5* | c.3341_3342del (p.K1114Rfs*10) / c.12499+1G>T | ES, GS | Commercial Carrier Screen |
| Ciliary dyskinesia, primary 40 | *DNAH9* | c.308del (p.F103Sfs*31) / c.958_959dup (p.K322Rfs*4) | ES, GS | None |
| Ciliary dyskinesia, primary 5 | *HYDIN* | c.11638G>A (p.D3880N) / c.3042+139A>G | GS | None |
| Citrullinemia Type 1 (n=2) | *ASS1* | c.1168G>A (p.G390R) / chr9:130438927-130445455 del (GRCh38)  c.1168G>A (p.G390R) (homozygous) | ES, GS | Commercial Carrier Screen |
| Coffin-Siris syndrome 2 | *ARID1A* | c.6200T>C (p.12067T) | ES, GS | None |
| COL1A1-related disorder | *COL1A1* | c.370-2A>T | ES, GS | Single-gene NIPT |
| COL4A1-related disorders (n=2) | *COL4A1* | c.3673G>A (p. G1225R)  c.1991-2A>G | ES, GS | None |
| COL7A1-related dystrophic epidermolysis bullosa (n=3) | *COL7A1* | c.6761G>A (p.G2254E) c.7025G>A (p.G2342D)  c.7234C>T (p.R2412*)/c.7796dup (p.S2600Ifs*28) | ES, GS | ACMG, Commercial Carrier Screen |
| Congenital anomalies of kidney and urinary tract 2 | *TBX18* | c.1045C>T (p.R349*) | ES, GS | None |
| Congenital heart defects, dysmorphic facial features, and intellectual developmental disorder | *CDK13* | c.484dup (p.Ala162GlyfsTer108) | ES, GS | None |
| Congenital heart defects, multiple types, 7 | *FLT4* | c.1755C>G (p.Y585*) | ES, GS | None |
| Congenital heart defects, nonsyndromic, 2 | *TAB2* | c.608_609insA (p.V204Cfs*3) | ES, GS | None |
| Cornelia de Lange syndrome 1 | *NIPBL* | c.2479_2480del (p.R827Gfs*2) | ES, GS | Single-gene NIPT |
| Cystic Fibrosis (n=5) | *CFTR* | c.1521_1523del (p.F508del) / c.3909C>G (p.N1303K)  **c.1521_1523del (p.F508del) (homozygous)**  c.1521_1523del (p.F508del) (homozygous)  **c.3846G>A (p.W1282*) /c.1521_1523del (p.F508del)**  c.1521_1523del (p.F508del) (homozygous) | ES, GS | ACOG, ACMG, Commercial Carrier Screen |
| Dehydrated hereditary stomatocytosis with or without pseudohyperkalemia and/or persistent edema | *PIEZO1* | c.7367G>A (p.R2456H) | ES, GS | None |
| Developmental and epileptic encephalopathy 43 | *GABRB3* | c.841A>G (p.Thr281Ala) | ES, GS | None |
| Developmental delay with or without dysmorphic facies and autism | *TRRAP* | c.8516_8559+67delinsAA | ES, GS | None |
| Developmental delay with variable intellectual disability and dysmorphic facies | *JARID2* | c.3379C>T (p.R1127*) | ES, GS | None |
| Diarrhea 1, secretory chloride, congenital | *SLC26A3* | c.614del (p.L205Rfs*28)/ c.670del (p.S224Pfs*9) | ES, GS | Commercial Carrier Screen |
| DYNC1H1 - related disorder | *DYNC1H1* | c.6148G>C (p.A2050P) | ES, GS | None |
| Epidermolytic hyperkeratosis | *KRT1* | c.564C>A (p.N188K) | ES, GS | None |
| Factor XI deficiency | *F11* | c.1778C>T (p.T593M) | ES, GS | Commercial Carrier Screen |
| FAM111B-related poikiloderma with multiple anomalies | *FAM111B* | c.1886T>G (p.F629C) | ES, GS | None |
| Familial focal epilepsy with variable foci 3 | *NPRL3* | chr16:98293-128265 dup(GRCh38) | CMA, ES, GS | None |
| Familial hypertrophic cardiomyopathy 27 | *ALPK3* | chr15:84826682-84830718 del (GRCh38) / c.4057G>C (p.G1353R) | ES, GS | None |
| Fraser syndrome | *FRAS1* | c.8922del (p.D2975Tfs*13) / c.4540+752A>G | GS | None |
| GATA4-related cardiac disorders | *GATA4* | c.796C>T (p.Arg266*) | ES, GS | None |
| GATA6-related disorder | *GATA6* | c.1367G>A (p.R456H) | ES, GS | None |
| Glucose/galactose malabsorption | *SLC5A1* | c.1281G>T (p.R427S) / c.1673G>A (p.R558H) | ES, GS | None |
| Glycerol kinase deficiency | *GK* | c.443dup (p.Y148*) | ES, GS | None |
| Glycine encephalopathy 1 | *GLDC* | c.1545G>C (p.R515S) / c.2665+1G>C | ES, GS | Commercial Carrier Screen |
| GNAS-related disorder | *GNAS* | c.432+1G>A | ES, GS | None |
| Helsmoortel-Van Der Aa syndrome | *ADNP* | c.-5-2A>G | ES, GS | None |
| Hemolytic anemia G6PD deficient (Favism) | *G6PD* | c.292G>A (p.V98M) | ES, GS | Commercial Carrier Screen |
| Hereditary hemorrhagic telangiectasia type 2 | *ACVRL1* | c.55dup (p.T19Nfs*19) | ES, GS | None |
| Hereditary nephrogenic diabetes insipidus | *AVPR2* | c.331_332del (p.Leu111Valfs*80) | ES, GS | Commercial Carrier Screen |
| Heteroptopia periventricular 1 | *FLNA* | c.7917_7918del (p.*2640Sfs*101) | ES, GS | None |
| HNF1B-related disorder | *HNF1B* | c.1130_1131del (p.Ser377Thrfs*Ter22) | ES, GS | None |
| HNF4A-related disorders | *HNF4A* | chr20:44395767-44406341 del (GRCh38) | CMA, ES, GS | None |
| Holoprosencephaly 9 | *GLI2* | c.2505del (p.S836Afs*76) | ES, GS | None |
| Holt-Oram syndrome | *TBX5* | c.408C>A (p.Tyr136*) | ES, GS | None |
| Ichthyosis, congenital, autosomal recessive 1 | *TGM1* | c.427C>T (p.R143C) / c.1314G>C (p.W438C) | ES, GS | None |
| Incontinentia pigmenti | *IKBKG* | exons 4-10 / X-inactivation ratio 86:14 | other - IKBKG single gene analysis | None |
| Intellectual developmental disorder autosomal dominant 26 | *AUTS2* | chr7:70714553-70806045 del (GRCh38) | CMA. ES. GS | None |
| Intellectual developmental disorder, autosomal dominant 23 | *SETD5* | c.2168T>A (p.L723*) | ES, GS | None |
| Intellectual developmental disorder, X-linked 93 | *BRWD3* | c.94dup (p.L32Pfs*43) | ES, GS | None |
| Intellectual developmental disorder, X-linked syndromic, Houge type | *CNKSR2* | chrX:21561750-21578871 del (GRCh38) | ES, GS | None |
| Isovaleric Acidemia | *IVD* | c.358C>T (p.Arg120*)(homozygous) | ES, GS | Commercial Carrier Screen |
| ITCH deficiency | *ITCH* | c.394dupA (homozygous) | ES, GS | None |
| Joubert/Meckel syndrome | *CC2D2* | c.3055C>T (p.R1019*) / c.3046G>A (p.E1016K) | ES, GS | None |
| Junctional epidermolysis bullosa | *LAMB3* | c.958_1034dup (p.N345Kfs*77) / c.1903C>T (p.R635*) | ES, GS | Commercial Carrier Screen |
| Kabuki syndrome (n=4) | *KMT2D* | c.4418+63T>G  c.3754C>T (p.R1252*) c.15079C>T (p.R5027*)  c.1634del (p.L545Rfs*385) | ES, GS | None |
| KAT6B-related neurodevelopmental disorder with multiple anomalies | *KAT6B* | c.798_799del (p.Ile267Argfs*9) | ES, GS | None |
| Kaufman oculocerebrofacial syndrome | *UBE3B* | c.2552del (p.E851Gfs*12) (homozygous) | ES, GS | None |
| KBG syndrome | *ANKRD11* | c.5364C>G (p.Y1788*) | ES, GS | None |
| Keratitis-Ichthyosis-Deafness syndrome | *GJB2* | c.263C>T (p.A88V) | ES, GS | None |
| Kleefstra syndrome 2 | *KMT2C* | c.6875del (p.P2292Hfs*13) | ES, GS | None |
| Laron syndrome | *GHR* | c.594A>G (p.E198=) (homozygous) | ES, GS | Commercial Carrier Screen |
| Long QT syndrome (n=3) | *KCNH2* | c.1682C>T (p.Ala561Val)  c.3106_3107dup (p.D1037Afs*21)  c.3106_3107dup (p.D1037Afs*21) | ES, GS | None |
| Maple syrup urine disease | *DBT* | c.871C>T (p.R291*) (homozygous) | ES, GS | Commercial Carrier Screen |
| Marfan syndrome | *FBN1* | c.3712 G>A (p.D1238N) | ES, GS | None |
| Mega-corpus-callosum syndrome with cerebellar hypoplasia and cortical malformations | *MAST1* | c.377C>A (p.T126K) | ES, GS | None |
| MEIS2-Related Disorder | *MEIS* | c.1030C>T (p.R344*) | ES, GS | None |
| Microcephaly, short stature, and limb anomalies | *DONSON* | c.[82A>C;1466A>C;c.786-33A>G] (p.[S28R;K489T]) / c.1047-9A>G | ES, GS | None |
| Mitochondrial complex 1 deficiency, ACAD9-Related | *ACAD9* | c.509C>T (p.A170V) / c.1807C>T (p.Q603*) | ES, GS | Commercial Carrier Screen |
| MT-AP6 -related disorders (n=2) | *MT-AP6* | m.9185T>C (p.L220P) m.650T>C (p.L217P) | None | None |
| Multiple endocrine neoplasia type 1 | *MEN1* | c.1220_1221del (p.P407Rfs*41) | ES, GS | None |
| Multiple pterygium syndrome (n=2) | *CHRNG* | c.402del (p.D135Tfs*48) / c.753_754del (p.V253Afs*44)  c.401_402del (p.P134Rfs*43) (homozygous) | ES, GS | Commercial Carrier Screen |
| MYH7-related disorders | *MYH7* | c.732+1del | ES, GS | None |
| Myotonic dystrophy type 1 | *DMPK* | CTG 5/1000 | GS, other - single gene PCR with reflex to southern blot | None |
| Nephrotic syndrome, type 1 | *NPHS1* | c.710T>C (p.L237P)(homozygous) | ES, GS | ACMG, Commercial Carrier Screen |
| Neurodevelopmental disorder with microcephaly, arthrogryposis, and structural brain anomalies | *SMPD4* | c.462+1G>T (homozygous) | ES, GS | None |
| NF1-related disorders (n=3) | *NF1* | c.2252-2A>G  c.3827G>A (p.R1276Q) c.7000-7_7000-2del | ES, GS | None |
| Noonan syndrome | *PTPN11* | c.329A>C (p.E110A) | ES, GS | Single-gene NIPT |
| Osteogenesis imperfecta (n=2) | *COL1A2, COL1A1* | c.2845G>A (p.G949S)  c.757C>T (p.Arg253*) | ES, GS | Single-gene NIPT |
| OTC deficiency | *OTC* | c.622G>A (p.A208T) | ES, GS | ACMG, Commercial Carrier Screen |
| Pyruvate dehydrogenase E1-alpha deficiency (n=2) | *PDHA1* | c.924_930dup (p.R311Gfs*5)  c.440A>T (p.K147I) | ES, GS | Commercial Carrier Screen |
| PIK3Ca-related overgrowth disorder | *PIK3CA* | c.1030G>A (p.V344M) | ES, GS | None |
| POR-related cytochrome P450 oxioreductase deficiency | *POR* | c.1825C>T (p.Q609*) / c.859G>C (p.A287P) | ES, GS | Commercial Carrier Screen |
| PPRT2-related disorder | *PPRT2* | c.483_487dup (p.Gln163Leufs*15) | ES, GS | None |
| Primary ciliary dyskinesia | *DNAH11* | c.13515_13526dup (p.Leu4507_Val4510dup) / c.6244C>T (p.Arg2082*) | ES, GS | Commercial Carrier Screen |
| Primary ciliary dyskinesia | *DNAH11* | c.6727C>T (p.R2243*) / c.13112C>T (p.P4371L) | ES, GS | None |
| Propionic acidemia, PCCB-related | *PCCB* | c.990dup (p.E331*) / c.975_977del (p.D325del) | ES, GS | Commercial Carrier Screen |
| PTPN11-related Disorders (n=3) | *PTPN11* | c.172A>G (p.N58D)  c.853T>C (p.F285L)  c.1492C>T (p.R498W) | ES, GS | Single-gene NIPT |
| Pulmonary hypertension, familial primary, 1, with or without HHT | *BMPR2* | c.1750C>T (p.R584*) | ES, GS | None |
| Pulmonary hypertension (n=2) | *SMAD9* | c.754delins38 (p.H252Cfs*41)   chr13:36871077-36880908 del (GRCh38) | ES, GS | None |
| Rett syndrome | *MECP2* | c.763C>T (p.R255*) | ES, GS | Single-gene NIPT, Commercial Carrier Screen |
| Rubinstein-Taybi syndrome 1 | *CREBBP* | c.5638C>T (p.Q1880*) | ES, GS | None |
| Saethre-Chotzen syndrome | *TWIST1* | chr7:19147961-19268007 del (GRCh37) | CMA, ES, GS | None |
| Schaaf-Yang syndrome | *MAGEL2* | c.1997del (p.Q666Rfs*36) | ES, GS | None |
| SCN2A-related disorders | *SCN2A* | c.4592A>C (p.Q1531P) | ES, GS | None |
| SCN8A-related disorder | *SCN8A* | c.2935_2936delinsAA (p.Ser979Asn) | ES, GS | None |
| Sickle cell anemia | *HBB* | c.20A>T (p.E7V) / c.-79A>G | GS, other - targeted gene testing | ACOG, ACMG, Commercial Carrier Screen |
| Sjogren-Larsson syndrome | *ALDH3A2* | c.1297_1298del (p.E433Rfs*3) / c.681-14T>A | ES, GS | Commercial Carrier Screen |
| Snijders Blok-Campeau syndrome | *CHD3* | c.5242C>T (p.R1748*) | ES, GS | None |
| Spherocytosis, type 2 | *SPTB* | chr14:64802670-64805317 del (GRCh38) | ES, GS | None |
| Spinal muscular atrophy (n=4) | *SMN1* | 0 copies | other | ACOG, ACMG, Commercial Carrier Screen |
| Stickler syndrome (n=2) | *COL2A1* | c.406del (p.D136lfs*63)  c.3436-1G>A | ES, GS | None |
| Stromme Syndrome | *CENPF* | c.3703C>T (p.Q1235*) /   c.171_199del (p.N57Kfs*11) | ES, GS | None |
| Supravalvar aortic stenosis | *ELN* | c.1747+2delinsGAGTTAG | ES, GS | None |
| Teebi hypertelorism syndrome 1 | *SPECC1L* | chr22:24299151-24319721 del (GRCh38) | ES, GS | None |
| Townes-Brocks syndrome | *SALL1* | c.1356del (p.R452Sfs*41) | ES, GS | None |
| Treacher Collins syndrome 1 | *TCOF1* | c.4169_4173del (p.A1390Efs*7) | ES, GS | None |
| TUBB-related complex cortical dysplasia with other brain malformations 6 | *TUBB* | c.244G>C (p.G82R) | ES, GS | None |
| Tuberous sclerosis | *TSC2* | chr16:2089751-2115694 del (GRCh37) | CMA, ES, GS | None |
| Tuberous sclerosis (n=3) | *TSC2* | c.4952A>G (p.N1651S)  c.4507C>T (p.Q1503*)  c.4489C>A (p.P1497T) | ES, GS | Single-gene NIPT |
| Vascular Ehlers Danlos syndrome | *COL3A1* | c.1979G>A (p.Gly660Asp) | ES, GS | None |
| Vitamin D Dependent Rickets, Type 1 | *CYP27B1* | c.1319_1325dup (p.F443Pfs*24) / c.1457G>C (p.R492P) | ES, GS | ACMG, Commercial Carrier Screen |
| Widermann-Steiner syndrome | *KMT2A* | c.1274dup (p.R426Sfs*7) | ES, GS | None |
| Witteveen-Kolk syndrome | *SIN3A* | c.1411C>T (p.R471*) | ES, GS | None |
| X-linked chondrodysplasia punctata 1 | *ARSL* | c.410G>C (p.G137A) | ES, GS | Commercial Carrier Screen |
| Zellweger spectrum disorders | *PEX1* | c.2097dup (p.I700Yfs*42) / c.2916del (p.G973Afs*16) | ES, GS | Commercial Carrier Screen |

Table S1: Unique diagnoses observed in the study cohort. Diagnoses observed more than once are indicated with (n= ) next to the diagnosis name; those without this notation were observed only once.

Beckwith-Wiedemann syndrome†, single gene analysis was not performed for these patients, only methylation studies

Abbreviations: FISH=fluorescence *in situ* hybridization; CMA=chromosomal microarray; ES= exome sequencing; GS=genome sequencing; NIPT=noninvasive prenatal test; SCA=sex chromosome aneuploidy; del=deletion; dup=duplication
