# Supplementary material for: Diagnosed After Birth—But Detectable Before? A Cohort Study of Prenatal Testing Potential
Source: Prenat Diagn. 2026 Jan 23;46(5-6):904–13. doi: 10.1002/pd.70072 (PMC13170046; doi:10.1002/pd.70072)
Supplement: Supplementary file 3 — Supporting Information S3 [file PD-46-904-s003.docx]

Supporting Information 3: Carrier Screening Panels

Table S1: ACOG Carrier Screen

| **Gene(s)** | **Condition Name** |
| --- | --- |
| CFTR | Cystic fibrosis |
| HBA1/HBA2 | Alpha-thalassemia |
| HBB | HBB-related hemoglobinopathies/thalassemias |
| SMN1 | Spinal muscular atrophy |

Table S2: ACMG Carrier Screen

| **Gene(s)** | **Condition Name** |
| --- | --- |
| ABCA3 | Surfactant metabolism dysfunction, pulmonary 3 |
| ABCC8 | Diabetes mellitus, permanent neonatal 3 |
| ABCD1 | Adrenoleukodystrophy (ALD) |
| ACADM | Medium-chain acyl-coenzyme A dehydrogenase deficiency |
| ACADVL | Very long chain acyl-CoA dehydrogenase deficiency |
| ACAT1 | ɑ-Methylacetoacetic aciduria |
| AFF2 | Mental retardation, X-linked, associated with fragile site FRAXE |
| AGA | Aspartylglucosaminuria |
| AGXT | Hyperoxaluria, primary type I |
| AHI1 | Joubert syndrome 3 |
| AIRE | Autoimmune polyendocrinopathy syndrome type I |
| ALDOB | Hereditary fructosuria |
| ALPL | Hypophosphatasia, adult; Hypophosphatasia, childhood and infantile |
| ANO10 | Spinocerebellar ataxia 10 |
| ARSA | Metachromatic leukodystrophy |
| ARX | Developmental and epileptic encephalopathy 1 (DEE1) |
| ASL | Argininosuccinate aciduria |
| ASPA | Canavan disease |
| ATP7B | Wilson disease |
| BBS1 | Bardet–Biedl syndrome 1 |
| BBS2 | Bardet–Biedl syndrome 2; Retinitis pigmentosa 74 |
| BCKDHB | Maple syrup urine disease |
| BLM | Bloom syndrome |
| BTD | Biotinidase deficiency |
| CBS | Homocystinuria, B6 responsive and nonresponsive |
| CC2D2A | Joubert syndrome 9; Meckel syndrome 6 |
| CCDC88C | Congenital hydrocephalus 1 |
| CEP290 | Joubert syndrome 5; Leber congenital amaurosis 10 |
| CFTR | Cystic fibrosis |
| CHRNE | Myasthenic syndrome, congenital, 4A, slow-channel; Myasthenic syndrome, congenital, 4B, fast-channe |
| CLCN1 | Congenital myotonia, autosomal recessive form |
| CLRN1 | Usher syndrome 3a |
| CNGB3 | Achromatopsia 3 |
| COL7A1 | Recessive dystrophic epidermolysis bullosa |
| CPT2 | Carnitine palmitoyltransferase II deficiency, infantile; Carnitine palmitoyltransferase II deficiency, lethal neonatal |
| CYP11A1 | Adrenal insufficiency, congenital, with 46, XY sex reversal, partial or complete |
| CYP21A2 | Congenital adrenal hyperplasia due to 21-hydroxylase deficiency |
| CYP27A1 | Cerebrotendinous xanthomatosis |
| CYP27B1 | Vitamin D–dependent rickets, type 1 |
| DHCR7 | Smith–Lemli–Opitz syndrome |
| DHDDS | Congenital disorder of glycosylation type 1; Retinitis pigmentosa 59 |
| DLD | Dihydrolipoamide dehydrogenase deficiency |
| DMD | Muscular dystrophy, Becker type (BMD); Muscular dystrophy, Duchenne type (DMD) |
| DYNC2H1 | Short-rib thoracic dysplasia 3 with or without polydactyly |
| ELP1 | Familial dysautonomia |
| ERCC2 | Cerebrooculofacioskeletal syndrome 2; Trichothiodystrophy 1, photosensitive |
| EVC2 | Chondroectodermal dysplasia |
| F8 | Hemophilia A (HEMA) |
| F9 | Hemophilia B (HEMB) |
| FAH | Tyrosinemia type I |
| FANCC | Fanconi anemia, complementation group C |
| FKRP | Muscular dystrophy–dystroglycanopathy, type A, 5; Muscular dystrophy–dystroglycanopathy, type B, 5 |
| FKTN | Cardiomyopathy, dilated, 1X; Walker–Warburg congenital muscular dystrophy |
| FMO3 | Trimethylaminuria |
| FMR1 | Fragile X syndrome (FXS) |
| FXN | Friedreich ataxia |
| G6PC | Glycogen storage disease type IA |
| GAA | Glycogen storage disease, type II (Pompe disease) |
| GALT | Galactosemia |
| GBA | Gaucher disease, type I; Gaucher disease, type II |
| GBE1 | Glycogen storage disease, type IV; GBE1-related disorders |
| GJB2 | Nonsyndromic hearing loss recessive 1A; Nonsyndromic hearing loss dominant 3A |
| GLA | Fabry disease |
| GNPTAB | Mucolipidosis type II alpha/beta; Mucolipidosis type III alpha/beta |
| GRIP1 | Fraser syndrome |
| HBA1/HBA2 | alpha-thalassemia |
| HBB | Sickle cell anemia β-thalassemia |
| HEXA | Tay–Sachs disease |
| HPS1 | Hermansky Pudlak S. 1 |
| HPS3 | Hermansky Pudlak S. 3 |
| IDUA | Mucopolysaccharidosis, Ih (Hurler S); Mucopolysaccharidosis, Ih/s (Hurler–Scheie S) |
| L1CAM | Hydrocephalus due to congenital stenosis of aqueduct of Sylvius (HSAS) |
| LRP2 | Donnai–Barrow syndrome |
| MCCC2 | 3-methylcrotonyl CoA carboxylase 2 deficiency |
| MCOLN1 | Mucolipidosis type IV |
| MCPH1 | Primary microcephaly 1, recessive |
| MID1 | Opitz GBBB syndrome, type I (GBBB1) |
| MLC1 | Megalencephalic leukoencephalopathy with subcortical cysts |
| MMACHC | Methylmalonic aciduria with homocystinuria cblC type |
| MMUT | Methylmalonic aciduria–methylmalonyl–CoA mutase deficiency |
| MVK | Hyper-IgD syndrome; Mevalonic aciduria |
| NAGA | Schindler disease, type 1 Schindler disease, type 3 |
| NEB | Nemaline myopathy 2 |
| NPHS1 | Finnish congenital nephrotic syndrome |
| NR0B1 | Adrenal hypoplasia, congenital (AHC) |
| OCA2 | Oculocutaneous albinism brown and type II |
| OTC | Ornithine transcarbamylase deficiency |
| PAH | Phenylketonuria |
| PCDH15 | Deafness, autosomal recessive 23; Usher syndrome, type 1F |
| PKHD1 | Autosomal recessive polycystic kidney disease |
| PLP1 | Spastic paraplegia 2, X-linked (SPG2) |
| PMM2 | Carbohydrate-deficient glycoprotein syndrome type Ia |
| POLG | Mitochondrial DNA depletion syndrome 4A; Mitochondrial DNA depletion syndrome 4B |
| PRF1 | Hemophagocytic lymphohistiocytosis, familial, 2 |
| RARS2 | Pontocerebellar hypoplasia type 6 |
| RNASEH2B | Aicardi Goutieres syndrome 2 |
| RPGR | Retinitis pigmentosa 3 (RP3; RP); Retinitis pigmentosa, X-linked, and sinorespiratory; Infections, with or without deafness; Macular degeneration, X-linked atrophic |
| RS1 | Retinoschisis 1, X-linked, juvenile (RS1) |
| SCO2 | Mitochondrial complex IV deficiency, nuclear type 2 |
| SLC19A3 | Basal ganglia disease, biotin-responsive |
| SLC26A2 | Epiphyseal dysplasia, multiple, 4; Achondrogenesis Ib |
| SLC26A4 | Deafness autosomal recessive 4; Pendred syndrome |
| SLC37A4 | Glycogen storage disease Ib; Glycogen storage disease Ic |
| SLC6A8 | Cerebral creatine deficiency syndrome 1 (CCDS1) |
| SMN1 | Spinal muscular atrophy |
| SMPD1 | Niemann–Pick disease, type A; Niemann–Pick disease, type B |
| TF | Atransferrinemia |
| TMEM216 | Joubert syndrome 2; Meckel syndrome 2 |
| TNXB | Ehlers–Danlos-like syndrome due to tenascin-X deficiency |
| TYR | Oculocutaneous albinism type 1A and 1B |
| USH2A | Usher syndrome, type 2A |
| XPC | Xeroderma pigmentosum |

Conditions included in ACMG Tier 3 carrier screening recommendation

Table S3: Commercial Carrier Screening Panel

| **Gene(s)** | **Condition Name** |
| --- | --- |
| AAAS | Triple A syndrome |
| ABAT | GABA-transaminase deficiency |
| ABCA12 | Autosomal recessive congenital ichthyosis (ABCA12-related) |
| ABCA3 | ABCA3-related conditions |
| ABCA4 | ABCA4-related conditions |
| ABCB11 | ABCB11-related conditions |
| ABCB4 | Progressive familial intrahepatic cholestasis 3 |
| ABCC2 | Dubin-Johnson syndrome |
| ABCC6 | Pseudoxanthoma Elasticum |
| ABCC8 | ABCC8-related conditions |
| ABCD1 | X-linked adrenoleukodystrophy |
| ACAD9 | Mitochondrial complex I deficiency 20/ACAD9 deficiency |
| ACADM | Medium-chain acyl-CoA dehydrogenase deficiency |
| ACADS | Short-Chain Acyl-CoA Dehydrogenase Deficiency |
| ACADVL | Very long-chain acyl-CoA dehydrogenase deficiency |
| ACAT1 | Beta-ketothiolase deficiency |
| ACOX1 | ACOX1-related conditions |
| ACSF3 | Combined malonic and methylmalonic aciduria |
| ADA | Adenosine deaminase deficiency |
| ADAMTS2 | Ehlers-Danlos syndrome, dermatosparaxis type |
| ADAMTSL4 | Isolated ectopia lentis |
| ADGRG1 | Polymicrogyria (ADGRG1-related) |
| ADGRV1 | ADGRV1-related conditions |
| AFF2 | Fragile XE Syndrome |
| AGA | Aspartylglucosaminuria |
| AGL | Glycogen storage disease type III |
| AGPS | Rhizomelic chondrodysplasia punctata type 3 |
| AGXT | Primary hyperoxaluria type 1 |
| AHI1 | AHI1-related conditions |
| AIFM1 | Combined oxidative phosphorylation deficiency 6 |
| AIPL1 | AIPL1-related conditions |
| AIRE | Autoimmune polyendocrinopathy with candidiasis and ectodermal dysplasia |
| ALDH3A2 | Sjögren-Larsson syndrome |
| ALDH7A1 | Pyridoxine-dependent epilepsy (ALDH7A1-related) |
| ALDOB | Hereditary fructose intolerance |
| ALG1 | Congenital disorder of glycosylation type Ik |
| ALG13 | ALG13-related conditions |
| ALG6 | Congenital disorder of glycosylation type Ic |
| ALMS1 | Alström syndrome |
| ALPL | Hypophosphatasia |
| AMN | Imerslund-Grsbeck syndrome |
| AMT | Glycine encephalopathy (AMT-related) |
| ANO10 | Spinocerebellar ataxia (ANO10-related) |
| AP1S1 | MEDNIK syndrome |
| AP3B1 | Hermansky-Pudlak syndrome 2 |
| AQP2 | Nephrogenic diabetes insipidus (AQP2-related) |
| AR | Androgen insensitivity syndrome |
| ARG1 | Arginase deficiency |
| ARL13B | Joubert syndrome 8 |
| ARL6 | ARL6-related conditions |
| ARSA | Metachromatic leukodystrophy (ARSA-related) |
| ARSB | Mucopolysaccharidosis type VI |
| ARSE | X-linked chondrodysplasia punctata type 1 |
| ARX | ARX-related conditions |
| ASAH1 | Farber lipogranulomatosis |
| ASL | Argininosuccinate lyase deficiency |
| ASNS | Asparagine synthetase deficiency |
| ASPA | Canavan disease |
| ASS1 | Citrullinemia type 1 |
| ATM | ATM-related conditions |
| ATP6V1B1 | Distal renal tubular acidosis with deafness (ATP6V1B1-related) |
| ATP7A | ATP7A-related conditions |
| ATP7B | Wilson disease |
| ATP8B1 | ATP8B1-related conditions |
| ATRX | Alpha-thalassemia X-linked intellectual disability syndrome |
| AVPR2 | AVPR2-related conditions |
| B9D1 | Joubert syndrome 27 |
| B9D2 | Joubert syndrome 34 |
| BBS1 | BBS1-related conditions |
| BBS10 | Bardet-Biedl syndrome (BBS10-related) |
| BBS12 | Bardet-Biedl syndrome (BBS12-related) |
| BBS2 | BBS2-related conditions |
| BBS4 | BBS4-related conditions |
| BBS5 | BBS5-related conditions |
| BBS7 | Bardet-Biedl syndrome (BBS7-related) |
| BBS9 | Bardet-Biedl syndrome (BBS9-related) |
| BCHE | Pseudocholinesterase Deficiency |
| BCKDHA | Maple syrup urine disease type 1A |
| BCKDHB | Maple syrup urine disease type 1B |
| BCS1L | BCS1L-related conditions |
| BLM | Bloom syndrome |
| BLOC1S3 | Hermansky-Pudlak syndrome type 8 |
| BLOC1S6 | Hermansky-Pudlak syndrome type 9 |
| BMP1 | Osteogenesis imperfecta (BMP1-related) |
| BRIP1 | BRIP1-related conditions |
| BSND | BSND-related conditions |
| BTD | Biotinidase deficiency |
| BTK | X-linked agammaglobulinemia |
| C2CD3 | Orofaciodigital syndrome XIV |
| CAD | Developmental and epileptic encephalopathy (CAD-related) |
| CANT1 | Desbuquois dysplasia type 1 |
| CAPN3 | Limb-girdle muscular dystrophy (CAPN3-related) |
| CASQ2 | Catecholaminergic polymorphic ventricular tachycardia (CASQ2-related) |
| CBS | Homocystinuria due to cystathionine beta-synthase deficiency |
| CC2D1A | Nonsyndromic intellectual disability (CC2D1A-related) |
| CC2D2A | CC2D2A-related conditions |
| CCDC103 | Primary ciliary dyskinesia (CCDC103-related) |
| CCDC39 | Primary ciliary dyskinesia (CCDC39-related) |
| CCDC88C | Congenital hydrocephalus-1 |
| CD3D | Severe combined immunodeficiency due to CD3-delta deficiency |
| CD3E | Severe combined immunodeficiency due to CD3-epsilon deficiency |
| CD40 | Hyper-IgM immunodeficiency (CD40-related) |
| CD40LG | X-linked hyper-IgM immunodeficiency |
| CD59 | Hemolytic anemia, CD59-mediated |
| CDH23 | CDH23-related conditions |
| CEP104 | Joubert syndrome 25 |
| CEP120 | Joubert syndrome 31; Short-rib thoracic dysplasia 13 with or without polydactyly |
| CEP152 | Seckel syndrome (CEP152-related) |
| CEP290 | CEP290-related conditions |
| CEP41 | Joubert syndrome 15 |
| CERKL | CERKL-related conditions |
| CFTR | CFTR-related conditions |
| CHAT | Congenital myasthenic syndrome (CHAT-related) |
| CHM | Choroideremia |
| CHRNE | Congenital myasthenic syndrome (CHRNE-related) |
| CHRNG | Multiple pterygium syndrome |
| CIB2 | Usher syndrome, type IJ; Deafness, autosomal recessive  48 |
| CIITA | Major histocompatibility complex class II deficiency (CIITA-related) |
| CLCN1 | Myotonia congenita |
| CLCN5 | Dent disease |
| CLN3 | CLN3-related conditions |
| CLN5 | Neuronal ceroid lipofuscinosis type 5 |
| CLN6 | Neuronal ceroid lipofuscinosis type 6 |
| CLN8 | Neuronal ceroid lipofuscinosis type 8 |
| CLRN1 | CLRN1-related conditions |
| CNGB3 | Achromatopsia (CNGB3-related) |
| COL11A2 | COL11A2-related conditions |
| COL17A1 | COL17A1-related conditions |
| COL27A1 | Steel syndrome |
| COL4A3 | Alport syndrome (COL4A3-related) |
| COL4A4 | Alport syndrome (COL4A4-related) |
| COL4A5 | Alport syndrome (COL4A5-related) |
| COL7A1 | Dystrophic epidermolysis bullosa |
| COLQ | Myasthenic syndrome, congenital, 5 |
| COX15 | Mitochondrial complex IV deficiency 6 |
| CPLANE1 | Joubert syndrome 17; Orofaciodigital syndrome VI |
| CPS1 | Carbamoyl phosphate synthetase I deficiency |
| CPT1A | Carnitine palmitoyltransferase I deficiency |
| CPT2 | Carnitine palmitoyltransferase II deficiency |
| CRB1 | CRB1-related conditions |
| CRTAP | Osteogenesis imperfecta (CRTAP-related) |
| CSPP1 | Joubert syndrome 21 |
| CTNS | Cystinosis |
| CTSA | Galactosialidosis |
| CTSC | CTSC-related conditions |
| CTSD | Neuronal ceroid lipofuscinosis type 10 |
| CTSK | Pycnodysostosis |
| CYBA | Chronic granulomatous disease (CYBA-related) |
| CYBB | Chronic granulomatous disease (CYBB-related) |
| CYP11A1 | Congenital adrenal insufficiency |
| CYP11B1 | CYP11B1-related conditions |
| CYP11B2 | Aldosterone synthase deficiency |
| CYP17A1 | CYP17A1-related conditions |
| CYP19A1 | Aromatase deficiency |
| CYP1B1 | CYP1B1-related conditions |
| CYP21A2 | Congenital adrenal hyperplasia due to 21-hydroxylase deficiency |
| CYP27A1 | Cerebrotendinous xanthomatosis |
| CYP27B1 | Vitamin D-dependent rickets type 1A |
| CYP7B1 | CYP7B1-related conditions |
| DBT | Maple syrup urine disease type 2 |
| DCAF17 | Woodhouse-Sakati syndrome |
| DCLRE1C | Severe combined immunodeficiency due to DCLRE1C (Artemis) deficiency |
| DCX | Miller-Dieker Syndrome |
| DDX11 | Warsaw syndrome |
| DFNB59 | PJVK-related conditions |
| DGAT1 | Congenital chronic diarrhea (DGAT1-related) |
| DGUOK | DGUOK-related conditions |
| DHCR7 | Smith-Lemli-Opitz syndrome |
| DHDDS | DHDDS-related conditions |
| DIS3L2 | Perlman Syndrome |
| DKC1 | Dyskeratosis congenita spectrum disorders (DKC1-related) |
| DLD | Dihydrolipoamide dehydrogenase deficiency |
| DLL3 | Spondylocostal dysostosis (DLL3-related) |
| DMD | DMD-related conditions |
| DNAH11 | Primary ciliary dyskinesia (DNAH11-related) |
| DNAH5 | Primary ciliary dyskinesia (DNAH5-related) |
| DNAI1 | Primary ciliary dyskinesia (DNAI1-related) |
| DNAI2 | Primary ciliary dyskinesia (DNAI2-related) |
| DNMT3B | Immunodeficiency-centromeric instability-facial anomalies syndrome 1 |
| DOK7 | DOK7-related conditions |
| DPYD | Dihydropyrimidine Dehydrogenase Deficiency; hereditary thymine-uraciluria |
| DUOX2 | DUOX2-related conditions |
| DYNC2H1 | DYNC2H1-related conditions |
| DYSF | DYSF-related conditions |
| EDA | EDA-related conditions |
| EIF2AK3 | Wolcott-Rallison syndrome |
| EIF2B1 | Leukoencephalopathy with vanishing white matter (EIF2B1-related) |
| EIF2B2 | Leukoencephalopathy with vanishing white matter (EIF2B2-related) |
| EIF2B3 | Leukoencephalopathy with vanishing white matter (EIF2B3-related) |
| EIF2B4 | Leukoencephalopathy with vanishing white matter (EIF2B4-related) |
| EIF2B5 | Leukoencephalopathy with vanishing white matter (EIF2B5-related) |
| ELP1 | Familial dysautonomia |
| EMD | Emery-Dreifuss muscular dystrophy (EMD-related) |
| EPG5 | Vici syndrome |
| ERCC2 | ERCC2-related conditions |
| ERCC6 | Cockayne syndrome B |
| ERCC8 | Cockayne syndrome A |
| ESCO2 | Roberts syndrome |
| ETFA | Glutaric acidemia type IIA |
| ETFB | Glutaric acidemia type IIB |
| ETFDH | Glutaric acidemia type IIC |
| ETHE1 | Ethylmalonic encephalopathy |
| EVC | Ellis-van Creveld syndrome (EVC-related) |
| EVC2 | EVC2-related conditions |
| EXOSC3 | Pontocerebellar hypoplasia type 1B |
| EYS | Retinitis pigmentosa 25 |
| F11 | Factor XI deficiency (hemophilia C) |
| F2 | Prothrombin-related thrombophilia (AD) |
| F5 | Factor V Leiden thrombophilia (AD) |
| F8 | Hemophilia A |
| F9 | Factor IX deficiency (hemophilia B) |
| FAH | Tyrosinemia type I |
| FAM161A | Retinitis pigmentosa 28 |
| FANCA | Fanconi anemia type A |
| FANCB | Fanconi anemia type B |
| FANCC | Fanconi anemia type C |
| FANCD2 | Fanconi anemia type D2 |
| FANCE | Fanconi anemia type E |
| FANCF | Fanconi anemia, complementation group F |
| FANCG | Fanconi anemia type G |
| FANCI | Fanconi anemia type I |
| FANCL | Fanconi anemia type L |
| FBP1 | Fructose-1,6-bisphosphatase deficiency |
| FBXO7 | Parkinson disease 15 |
| FH | FH-related conditions |
| FHL1 | FHL1-related conditions |
| FKBP10 | FKBP10-related conditions |
| FKRP | Muscular dystrophy-dystroglycanopathy (FKRP-related) |
| FKTN | Muscular dystrophy-dystroglycanopathy (FKTN-related) |
| FMO3 | Trimethylaminuria |
| FMR1 | FMR1-related conditions including fragile X syndrome |
| FOXN1 | FOXN1-related conditions |
| FOXP3 | Immunodysregulation, polyendocrinopathy, and enteropathy, X-linked |
| FOXRED1 | Mitochondrial complex I deficiency 19 |
| FRAS1 | Fraser syndrome (FRAS1-related) |
| FREM2 | Fraser syndrome (FREM2-related) |
| FUCA1 | Fucosidosis |
| FXN | Friedreich Ataxia |
| G6PC | Glycogen storage disease type Ia |
| G6PC3 | Severe congenital neutropenia due to G6PC3 deficiency |
| G6PD | Glucose-6-phosphate dehydrogenase deficiency |
| GAA | Glycogen storage disease type II (Pompe disease) |
| GALC | Krabbe disease |
| GALE | Epimerase deficiency galactosemia |
| GALK1 | Galactokinase deficiency galactosemia |
| GALNS | Mucopolysaccharidosis type IVA |
| GALNT3 | Hyperphosphatemic familial tumoral calcinosis (GALNT3-related) |
| GALT | Galactosemia (GALT-related) |
| GAMT | Guanidinoacetate methyltransferase deficiency |
| GATM | GATM-related conditions |
| GBA | GBA-related conditions; Gaucher disease |
| GBE1 | GBE1-related conditions |
| GCDH | Glutaric acidemia type I |
| GCH1 | GCH1-related conditions |
| GDF5 | GDF5-related conditions |
| GFM1 | Combined oxidative phosphorylation deficiency 1 |
| GHR | GHR-related conditions |
| GJB1 | Charcot-Marie-Tooth disease type 1X |
| GJB2 | GJB2-related conditions |
| GLA | Fabry disease |
| GLB1 | GLB1-related conditions |
| GLDC | Glycine encephalopathy (GLDC-related) |
| GLE1 | GLE1-related conditions |
| GNE | GNE-related conditions |
| GNPAT | Rhizomelic chondrodysplasia punctata type 2 |
| GNPTAB | GNPTAB-related conditions |
| GNPTG | Mucolipidosis type III gamma |
| GNS | Mucopolysaccharidosis type IIID |
| GORAB | Geroderma osteodysplastica |
| GP1BA | GP1BA-related conditions |
| GP9 | Bernard-Soulier syndrome (GP9-related) |
| GRHPR | Primary hyperoxaluria type 2 |
| GRIP1 | Fraser syndrome (GRIP1-related) |
| GSS | Glutathione synthetase deficiency |
| GUCY2D | GUCY2D-related conditions |
| GUSB | Mucopolysaccharidosis type VII |
| HADH | Medium/short-chain 3-hydroxyacyl-CoA dehydrogenase deficiency |
| HADHA | HADHA-related conditions |
| HADHB | Mitochondrial trifunctional protein deficiency (HADHB-related) |
| HAMP | Hereditary hemochromatosis type 2 (HAMP-related) |
| HAX1 | Severe congenital neutropenia due to HAX1 deficiency |
| HBA1/HBA2 | Alpha-thalassemia |
| HBB | HBB-related hemoglobinopathies |
| HCFC1 | HCFC1-related conditions |
| HEXA | Tay-Sachs disease |
| HEXB | Sandhoff disease |
| HFE | Hereditary hemochromatosis type 1 |
| HGD | Alkaptonuria |
| HGSNAT | HGSNAT-related conditions |
| HJV/HFE2 | Hereditary hemochromatosis type 2 (HJV-related) |
| HLCS | Holocarboxylase synthetase deficiency |
| HMGCL | 3-hydroxy-3-methylglutaryl-CoA lyase deficiency |
| HMOX1 | Heme oxygenase 1 deficiency |
| HOGA1 | Primary hyperoxaluria type 3 |
| HPD | Tyrosinemia type III |
| HPRT1 | HPRT1-related conditions |
| HPS1 | Hermansky-Pudlak syndrome type 1 |
| HPS3 | Hermansky-Pudlak syndrome type 3 |
| HPS4 | Hermansky-Pudlak syndrome type 4 |
| HPS5 | Hermansky-Pudlak syndrome type 5 |
| HPS6 | Hermansky-Pudlak syndrome type 6 |
| HSD17B10 | 2-methyl-3-hydroxybutyric aciduria |
| HSD17B3 | 17-beta hydroxysteroid dehydrogenase 3 deficiency |
| HSD17B4 | HSD17B4-related conditions |
| HSD3B2 | Congenital adrenal hyperplasia due to 3-beta-hydroxysteroid dehydrogenase deficiency |
| HYAL1 | Mucopolysaccharidosis type IX |
| HYLS1 | Hydrolethalus syndrome type 1 |
| IDS | Mucopolysaccharidosis type II |
| IDUA | Mucopolysaccharidosis type I |
| IGHMBP2 | IGHMBP2-related conditions |
| IKBKB | IKBKB-related conditions |
| IL2RG | X-linked severe combined immunodeficiency |
| IL7R | Severe combined immunodeficiency due to IL7R-alpha deficiency |
| INPP5E | Joubert Syndrome |
| INVS | Nephronophthisis (INVS-related) |
| IQCB1 | Senior-Loken syndrome 5 |
| ISPD/CRPPA | Muscular dystrophy-dystroglycanopathy (congenital with brain and eye anomalies), type A, 7; Muscular dystrophy- dystroglycanopathy (limb-girdle), type C, 7 |
| ITGA6 | Junctional epidermolysis bullosa with pyloric atresia (ITGA6-related) |
| ITGB3 | ITGB3-related conditions |
| ITGB4 | Epidermolysis bullosa with pyloric atresia (ITGB4-related) |
| IVD | Isovaleric acidemia |
| JAK3 | Severe combined immunodeficiency due to JAK3 deficiency |
| KCNJ1 | Bartter syndrome type 2 |
| KCNJ11 | KCNJ11-related conditions |
| L1CAM | L1 syndrome |
| LAMA2 | LAMA2-related muscular dystrophy |
| LAMA3 | LAMA3-related conditions |
| LAMB3 | LAMB3-related conditions |
| LAMC2 | Junctional epidermolysis bullosa (LAMC2-related) |
| LARGE1 | Muscular dystrophy-dystroglycanopathy (LARGE1-related) |
| LCA5 | Leber congenital amaurosis 5 |
| LDLR | Familial hypercholesterolemia (LDLR-related) (AD) |
| LDLRAP1 | Familial hypercholesterolemia (LDLRAP1-related) |
| LHX3 | Combined pituitary hormone deficiency (LHX3-related) |
| LIFR | Stüve-Wiedemann syndrome |
| LIG4 | LIG4 syndrome |
| LIPA | Lysosomal acid lipase deficiency |
| LMBRD1 | Cobalamin F deficiency |
| LOXHD1 | Nonsyndromic deafness (LOXHD1-related) |
| LPL | Familial chylomicronemia syndrome |
| LRAT | LRAT-related conditions |
| LRP2 | Donnai-Barrow syndrome |
| LRPPRC | Mitochondrial complex IV deficiency / Leigh syndrome, French Canadian type |
| LYST | Chediak-Higashi syndrome |
| MAK | Retinitis pigmentosa 62 |
| MAN2B1 | Alpha-mannosidosis |
| MANBA | Beta-mannosidosis |
| MCCC1 | 3-methylcrotonyl-CoA carboxylase (3-MCC) deficiency (MCCC1-related) |
| MCCC2 | 3-methylcrotonyl-CoA carboxylase (3-MCC) deficiency (MCCC2-related) |
| MCEE | Methylmalonic acidemia (MCEE-related) |
| MCOLN1 | Mucolipidosis type IV |
| MCPH1 | Primary microcephaly (MCPH1-related) |
| MECP2 | MECP2-related conditions |
| MECR | Childhood-onset dystonia with optic atrophy and basal ganglia abnormalities |
| MED17 | Microcephaly, postnatal progressive, with seizures and brain atrophy |
| MEFV | Familial Mediterranean fever |
| MESP2 | Spondylocostal dysostosis (MESP2-related) |
| MFSD8 | MFSD8-related conditions |
| MID1 | Opitz GBBB syndrome (MID1-related) |
| MKKS | MKKS-related conditions |
| MKS1 | Joubert syndrome and related disorders (MKS1-related) |
| MLC1 | Megalencephalic leukoencephalopathy with subcortical cysts 1 |
| MLYCD | Malonyl-CoA decarboxylase deficiency |
| MMAA | Methylmalonic acidemia (MMAA-related) |
| MMAB | Methylmalonic acidemia (MMAB-related) |
| MMACHC | Cobalamin C deficiency |
| MMADHC | Cobalamin D deficiency |
| MOCS1 | Molybdenum cofactor deficiency (MOCS1-related) |
| MOCS2 | Molybdenum cofactor deficiency (MOCS2-related) |
| MPI | Congenital disorder of glycosylation type Ib |
| MPL | MPL-related conditions |
| MPV17 | MPV17-related conditions |
| MRE11 | Ataxia-telangiectasia-like disorder |
| MTHFR | Homocystinuria due to MTHFR deficiency |
| MTM1 | X-linked myotubular myopathy |
| MTR | Homocystinuria due to cobalamin G deficiency |
| MTRR | Homocystinuria due to cobalamin E deficiency |
| MTTP | Abetalipoproteinemia |
| MUSK | MUSK-related conditions |
| MUT | Methylmalonic acidemia (MUT-related) |
| MVK | MVK-related conditions |
| MYO15A | Nonsyndromic deafness (MYO15A-related) |
| MYO7A | MYO7A-related conditions |
| NAGA | Alpha-N-acetylgalactosaminidase deficiency |
| NAGLU | Mucopolysaccharidosis type IIIB |
| NAGS | N-acetylglutamate synthase deficiency |
| NBN | Nijmegen breakage syndrome |
| NCF2 | Chronic granulomatous disease (NCF2-related) |
| NDRG1 | Charcot-Marie-Tooth disease type 4D |
| NDUFAF2 | Mitochondrial complex I deficiency 10 |
| NDUFAF5 | Mitochondrial complex I deficiency 16 |
| NDUFAF6 | Molybdenum cofactor deficiency A |
| NDUFS4 | Mitochondrial complex I deficiency 1 |
| NDUFS6 | Mitochondrial complex I deficiency 9 |
| NDUFS7 | Mitochondrial complex I deficiency 3 |
| NDUFV1 | Mitochondrial complex I deficiency 4 |
| NEB | Nemaline myopathy 2 |
| NEU1 | Sialidosis |
| NGLY1 | Congenital disorder of glycosylation type Iv |
| NPC1 | Niemann-Pick disease type C (NPC1-related) |
| NPC2 | Niemann-Pick disease type C (NPC2-related) |
| NPHP1 | Nephronophthisis (NPHP1-related) |
| NPHP3 | Juvenile Nephronophthisis; Renal-Hepatic-Pancreatic  Dysplasia; RHPD |
| NPHP4 | Senior-Loken syndrome 4 |
| NPHS1 | Congenital nephrotic syndrome type 1 |
| NPHS2 | Congenital nephrotic syndrome type 2 |
| NR0B1 | NR0B1-related conditions |
| NR2E3 | NR2E3-related conditions |
| NSMCE3 | NSMCE3 deficiency |
| NTRK1 | Congenital insensitivity to pain with anhidrosis |
| OAT | Gyrate atrophy of the choroid and retina |
| OCA2 | Oculocutaneous albinism type 2 |
| OCRL | OCRL-related conditions |
| OPA3 | OPA3-related conditions |
| OSTM1 | OSTM1 deficiency associated osteopetrosis |
| OTC | Ornithine transcarbamylase deficiency |
| OTOA | Nonsyndromic deafness (OTOA-related) |
| OTOF | OTOF-related conditions |
| P3H1 | Osteogenesis imperfecta (P3H1-related) |
| PAH | Phenylalanine hydroxylase deficiency |
| PANK2 | Pantothenate kinase-associated neurodegeneration |
| PC | Pyruvate carboxylase deficiency |
| PCBD1 | Biopterin-deficient hyperphenylalaninemia (PCBD1-related) |
| PCCA | Propionic acidemia (PCCA-related) |
| PCCB | Propionic acidemia (PCCB-related) |
| PCDH15 | PCDH15-related conditions |
| PCNT | Microcephalic osteodysplastic primordial dwarfism type II |
| PDHA1 | Pyruvate dehydrogenase complex deficiency (PDHA1-related) |
| PDHB | Pyruvate dehydrogenase complex deficiency (PDHB-related) |
| PEPD | Prolidase deficiency |
| PET100 | Mitochondrial complex IV deficiency 12 |
| PEX1 | Zellweger spectrum disorder (PEX1-related) |
| PEX10 | Zellweger spectrum disorder (PEX10-related) |
| PEX12 | Zellweger spectrum disorder (PEX12-related) |
| PEX13 | Zellweger spectrum disorder (PEX13-related) |
| PEX16 | Zellweger spectrum disorder (PEX16-related) |
| PEX2 | Zellweger spectrum disorder (PEX2-related) |
| PEX26 | Zellweger spectrum disorder (PEX26-related) |
| PEX5 | PEX5-related conditions |
| PEX6 | Zellweger spectrum disorder (PEX6-related) |
| PEX7 | PEX7-related conditions |
| PFKM | Glycogen storage disease type VII |
| PGM3 | PGM3-congenital disorder of glycosylation |
| PHGDH | Phosphoglycerate dehydrogenase deficiency |
| PHKB | Glycogen storage disease type IXb |
| PHKG2 | Glycogen storage disease type IXc |
| PHYH | Refsum disease (PHYH-related) |
| PIGN | PIGN-congenital disorder of glycosylation |
| PKHD1 | Polycystic kidney disease (PKHD1-related) |
| PLA2G6 | PLA2G6-related conditions |
| PLCE1 | Nephrotic syndrome |
| PLEKHG5 | PLEKHG5-related conditions |
| PLOD1 | Ehlers-Danlos syndrome, kyphoscoliotic type |
| PLP1 | PLP1-related conditions |
| PMM2 | Congenital disorder of glycosylation type Ia |
| PNPO | Pyridoxal 5'-phosphate-dependent epilepsy |
| POLG | POLG-related conditions |
| POLH | Xeroderma pigmentosum, variant type |
| POMGNT1 | POMGNT1-related conditions |
| POMT1 | Muscular dystrophy-dystroglycanopathy (POMT1-related) |
| POMT2 | Muscular dystrophy-dystroglycanopathy (POMT2-related) |
| POR | Cytochrome P450 oxidoreductase deficiency |
| POU1F1 | Combined pituitary hormone deficiency (POU1F1-related) |
| PPT1 | Neuronal ceroid lipofuscinosis type 1 |
| PRCD | Retinitis pigmentosa 36 |
| PRDM5 | Brittle cornea syndrome (PRDM5-related) |
| PRF1 | Familial hemophagocytic lymphohistiocytosis type 2 |
| PROP1 | Combined pituitary hormone deficiency (PROP1-related) |
| PRPS1 | PRPS1-related conditions |
| PSAP | PSAP-related conditions |
| PTPRC | Severe combined immunodeficiency due to CD45 deficiency |
| PTS | Biopterin-deficient hyperphenylalaninemia (PTS-related) |
| PUS1 | Myopathy, lactic acidosis, and sideroblastic anemia 1 |
| PYGM | Glycogen storage disease type V |
| QDPR | Biopterin-deficient hyperphenylalaninemia (QDPR-related) |
| RAB23 | Carpenter syndrome (RAB23-related) |
| RAG1 | Severe combined immunodeficiency due to RAG1 deficiency |
| RAG2 | Severe combined immunodeficiency due to RAG2 deficiency |
| RAPSN | RAPSN-related conditions |
| RARS2 | Pontocerebellar hypoplasia type 6 |
| RDH12 | RDH12-related conditions |
| RLBP1 | RLBP1-related conditions |
| RMRP | Cartilage-hair hypoplasia-anauxetic dysplasia spectrum disorders |
| RNASEH2A | Aicardi-Goutieres syndrome 4 |
| RNASEH2B | Aicardi-Goutieres syndrome 2 |
| RNASEH2C | Aicardi-Goutieres syndrome 3 |
| RP2 | Retinitis pigmentosa 2 |
| RPE65 | RPE65-related conditions |
| RPGR | Retinitis Pigmentosa, X-Linked, RPGR-Related |
| RPGRIP1L | Joubert syndrome and related disorders (RPGRIP1L-related) |
| RS1 | X-linked juvenile retinoschisis |
| RTEL1 | Dyskeratosis congenita spectrum disorders (RTEL1-related) |
| RXYLT1 | Muscular dystrophy-dystroglycanopathy (RXYLT1-related) |
| RYR1 | RYR1-related conditions |
| SACS | Autosomal recessive spastic ataxia of Charlevoix-Saguenay |
| SAMD9 | SAMD9-related conditions |
| SAMHD1 | Aicardi-Goutieres syndrome 5 |
| SBDS | Shwachman-Diamond syndrome |
| SCARB2 | Action Myoclonus – Renal Failure Syndrome |
| SCO2 | Cardioencephalomyopathy |
| SEC23B | Congenital dyserythropoietic anemia type II |
| SEPSECS | Pontocerebellar hypoplasia type 2D |
| SERPINA1 | Alpha-1 antitrypsin deficiency |
| SGCA | Limb-girdle muscular dystrophy type 2D |
| SGCB | Limb-girdle muscular dystrophy type 2E |
| SGCD | Limb-girdle muscular dystrophy type 2F |
| SGCG | Limb-girdle muscular dystrophy type 2C |
| SGSH | Mucopolysaccharidosis type IIIA |
| SKIV2L | Trichohepatoenteric syndrome (SKIV2L-related) |
| SLC12A1 | Bartter syndrome type 1 |
| SLC12A3 | Gitelman syndrome |
| SLC12A6 | SLC12A6-related conditions |
| SLC17A5 | Sialic acid storage diseases |
| SLC19A2 | Thiamine-responsive megaloblastic anemia |
| SLC19A3 | Biotin-responsive basal ganglia disease |
| SLC1A4 | Spastic tetraplegia, thin corpus callosum, and progressive microcephaly |
| SLC22A5 | Primary carnitine deficiency |
| SLC25A13 | Citrin deficiency |
| SLC25A15 | Hyperornithinemia-hyperammonemia-homocitrullinuria syndrome |
| SLC25A20 | Carnitine-acylcarnitine translocase deficiency |
| SLC26A2 | SLC26A2-related conditions |
| SLC26A3 | Congenital secretory chloride diarrhea |
| SLC26A4 | SLC26A4-related conditions |
| SLC27A4 | Ichthyosis prematurity syndrome |
| SLC35A3 | Congenital disorder of glycosylation (SLC35A3-related) |
| SLC37A4 | SLC37A4-related conditions |
| SLC38A8 | Foveal hypoplasia (SLC38A8-related) |
| SLC39A4 | Acrodermatitis enteropathica |
| SLC45A2 | Oculocutaneous albinism type 4 |
| SLC4A11 | Corneal dystrophy and perceptive deafness |
| SLC4A4 | Renal tubular acidosis |
| SLC5A5 | Thyroid dyshormonogenesis (SLC5A5-related) |
| SLC6A8 | X-linked creatine transporter deficiency |
| SLC7A7 | Lysinuric protein intolerance |
| SMARCAL1 | Schimke immuno-osseous dysplasia |
| SMN1 | Spinal muscular atrophy  Carrier residual risks listed are for 2 copy SMN1 results. Carrier residual risk for >2 copies are 5- to 10-fold lower. |
| SMPD1 | Niemann-Pick disease types A and B |
| SNAP29 | Cerebral dysgenesis, neuropathy, ichthyosis, and keratoderma |
| SPG11 | SPG11-related conditions |
| SPR | Sepiapterin reductase deficiency |
| SRD5A2 | Steroid 5-alpha-reductase deficiency |
| ST3GAL5 | GM3 synthase deficiency |
| STAR | Lipoid congenital adrenal hyperplasia |
| STX11 | Familial hemophagocytic lymphohistiocytosis type 4 |
| STXBP2 | Familial hemophagocytic lymphohistiocytosis type 5 |
| SUMF1 | Multiple sulfatase deficiency |
| SUOX | Sulfite oxidase deficiency |
| SURF1 | SURF1-related conditions |
| SYNE4 | Nonsyndromic deafness (SYNE4-related) |
| TANGO2 | Metabolic crises with rhabdomyolysis, cardiac arrhythmias and neurodegeneration |
| TAT | Tyrosinemia type II |
| TAZ | Barth syndrome |
| TBCD | Progressive early-onset encepahlopathy with brain atrophy and thin corpus callosum (PEBAT) |
| TBCE | TBCE-related conditions |
| TCIRG1 | Osteopetrosis (TCIRG1-related) |
| TCN2 | Transcobalamin II deficiency |
| TECPR2 | Spastic paraplegia type 49 |
| TERT | Dyskeratosis congenita spectrum disorders (TERT-related) |
| TF | Atransferrinemia |
| TFR2 | Hereditary hemochromatosis type 3 |
| TG | Thyroid dyshormonogenesis (TG-related) |
| TGM1 | Autosomal recessive congenital ichthyosis (TGM1-related) |
| TH | Tyrosine hydroxylase deficiency |
| TJP2 | Cholestasis, progressive familial intrahepatic 4; Hypercholanemia, familial |
| TK2 | Mitochondrial DNA depletion syndrome-2 |
| TMC1 | Nonsyndromic deafness (TMC1-related) |
| TMEM216 | Joubert syndrome and related disorders (TMEM216-related) |
| TMEM67 | TMEM67-related conditions |
| TMPRSS3 | Nonsyndromic deafness (TMPRSS3-related) |
| TNXB | Ehlers-Danlos Syndrome, Classic-Like, TNXB-Related |
| TPO | Thyroid dyshormonogenesis (TPO-related) |
| TPP1 | Neuronal ceroid lipofuscinosis type 2 |
| TREX1 | TREX1-related conditions |
| TRIM32 | TRIM32-related conditions |
| TRIM37 | Mulibrey nanism |
| TRMU | Transient infantile liver failure |
| TSEN2 | Pontocerebellar hypoplasia (TSEN54-related) |
| TSEN54 | Pontocerebellar Hypoplasia; Pontocerebellar Hypoplasia 4; PCH4; Pontocerebellar Hypoplasia 2A; PCH2A |
| TSFM | Combined oxidative phosphorylation deficiency 3 |
| TSHB | Congenital hypothyroidism (TSHB-related) |
| TSHR | TSHR-related conditions |
| TTC37 | Trichohepatoenteric syndrome (TTC37-related) |
| TTC8 | Bardet-Biedl syndrome 8 |
| TTPA | Ataxia with vitamin E deficiency |
| TULP1 | TULP1-related conditions |
| TYMP | Mitochondrial neurogastrointestinal encephalomyopathy |
| TYR | Oculocutaneous albinism types 1A and 1B |
| TYRP1 | Oculocutaneous albinism type 3 |
| UBA1 | Spinal muscular atrophy, X-linked 2, infantile |
| UBR1 | Johanson-Blizzard syndrome |
| UNC13D | Familial hemophagocytic lymphohistiocytosis type 3 |
| UPB1 | Beta-ureidopropionase deficiency |
| USH1C | USH1C-related conditions |
| USH2A | USH2A-related conditions |
| VDR | Vitamin D-dependent rickets type 2A |
| VLDLR | Cerebellar ataxia, intellectual disability, and dysequilibrium syndrome 1 |
| VPS11 | Hypomyelinating leukodystrophy-12 |
| VPS13A | Chorea-acanthocytosis |
| VPS13B | Cohen syndrome |
| VPS45 | Severe congenital neutropenia due to VPS45 deficiency |
| VPS53 | VPS53-related conditions |
| VRK1 | VRK1-related conditions |
| VSX2 | VSX2-related conditions |
| WAS | WAS-related conditions |
| WISP3 | Progressive pseudorheumatoid dysplasia |
| WNT10A | WNT10A-related conditions |
| WRN | Werner syndrome |
| WWOX | Spinocerebellar ataxia-12 |
| XPA | Xeroderma pigmentosum complementation group A |
| XPC | Xeroderma pigmentosum complementation group C |
| ZBTB24 | Immunodeficiency-centromeric instability-facial anomalies syndrome 2 |
| ZFYVE26 | Spastic paraplegia type 15 |
| ZIC3 | Heterotaxy; Heterotaxy, Visceral, 1, XLR; HTX1 |
| ZNF469 | Brittle cornea syndrome (ZNF469-related) |

Table S3 includes the 614 conditions included in the “commercial carrier screening panel”, comprised of the number of unique conditions between two commercially available carrier screening panels (Myriad Foresight 267 gene panel and Natera Horizon 613 gene panel)
